# Senescent hepatic stellate cells promote liver regeneration through IL-6 and ligands of CXCR2

**DOI:** 10.1172/jci.insight.158207

**Published:** 2022-06-16

**Authors:** Naiyuan Cheng, Ki-Hyun Kim, Lester F. Lau

**Affiliations:** Department of Biochemistry and Molecular Genetics, University of Illinois at Chicago College of Medicine, Chicago, Illinois, USA.

**Keywords:** Hepatology, Cellular senescence, Cytokines, Integrins

## Abstract

Senescent cells have long been associated with deleterious effects in aging-related pathologies, although recent studies have uncovered their beneficial roles in certain contexts, such as wound healing. We have found that hepatic stellate cells (HSCs) underwent senescence within 2 days after 2/3 partial hepatectomy (PHx) in young (2–3 months old) mice, and the elimination of these senescent cells by using the senolytic drug ABT263 or by using a genetic mouse model impaired liver regeneration. Senescent HSCs secrete IL-6 and CXCR2 ligands as part of the senescence-associated secretory phenotype, which induces multiple signaling pathways to stimulate liver regeneration. IL-6 activates STAT3, induces Yes-associated protein (YAP) activation through SRC family kinases, and synergizes with CXCL2 to activate ERK1/2 to stimulate hepatocyte proliferation. The administration of either IL-6 or CXCL2 partially restored liver regeneration in mice with senescent cell elimination, and the combination of both fully restored liver weight recovery. Furthermore, the matricellular protein central communication network factor 1 (CCN1, previously called CYR61) was rapidly elevated in response to PHx and induced HSC senescence. Knockin mice expressing a mutant CCN1 unable to bind integrin α_6_β_1_ were deficient in senescent cells and liver regeneration after PHx. Thus, HSC senescence, largely induced by CCN1, is a programmed response to PHx and plays a critical role in liver regeneration through signaling pathways activated by IL-6 and ligands of CXCR2.

## Graphical abstract

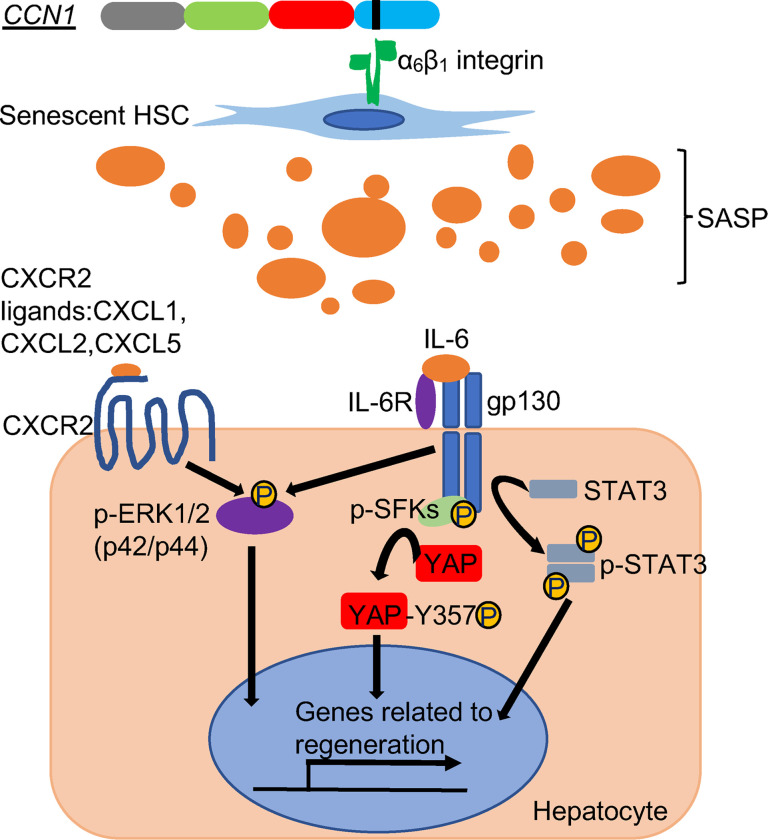

## Introduction

The adult liver possesses remarkable regenerative capacity. Upon acute injury or significant loss of tissue, the liver can grow rapidly to regenerate the original organ size through a combination of hepatocyte proliferation and hypertrophy to maintain homeostasis (1, 2). This unique and robust regenerative function allows surgical resection and split liver transplantation from living donors as treatment options for advanced liver diseases and certain liver cancers (3). The mechanism of liver regeneration has been studied extensively in rodents using 2/3 partial hepatectomy (PHx) as an experimental model in which the resected liver is completely regenerated within 7–14 days (1, 2). Extensive gene expression changes occur in hepatocytes following PHx, with many genes being rapidly activated (4, 5). Multiple complementary and compensatory signaling pathways contribute to liver regeneration, and the inactivation of any single pathway delays but does not eliminate regeneration (2). Despite accumulating information on the mechanism of liver regeneration, the complex regulatory networks that control diverse facets of this process remain incompletely understood.

Cellular senescence is a form of irreversible cell cycle arrest triggered by stress-induced DNA damage responses (6, 7). Paradoxically, senescence can evoke both beneficial and deleterious effects in a context-dependent manner. For example, cellular senescence can be a powerful mechanism of tumor suppression, yet it can also promote tumorigenesis in some circumstances (8–10). Chronic or persistent senescence, which may occur due to sustained environmental insults, cellular stresses, and accumulated macromolecular damages, is associated with harmful effects on organ functions and exacerbates aging (11–13). By contrast, acute or transient senescence may be induced by paracrine signals during development or wound healing and may play beneficial roles (14–17). Many of the effects of senescent cells are mediated through the expression of the senescence-associate secretory phenotype (SASP), which may vary from cell type to cell type but in general alters the cellular microenvironment by the secretion of proinflammatory chemokines, cytokines, growth factors, and proteases (7, 18). In the liver, recent studies have found that cellular senescence in parenchymal cells can be detrimental to regeneration (19, 20), but senescence in hepatic stellate cells (HSCs) may limit and reduce fibrosis (21, 22).

To minimize the effect of age-related chronic senescence in PHx, we have focused on sexually mature young (2–3 months old) mice. We found that senescent HSCs were rapidly induced in the liver after PHx, triggered primarily by the matricellular protein central communication network factor 1 (CCN1, previously named CYR61) (23, 24). Remarkably, the elimination of these senescent cells in young mice using either pharmacological or genetic approaches impaired liver weight restoration and hepatocyte proliferation. Optimal liver regeneration after PHx required IL-6 and CXCR2 ligands expressed by senescent HSCs as part of the SASP. These findings show that the acute accumulation of senescent HSCs is a programmed response to PHx regulated by CCN1 and plays a key role in promoting liver regeneration through signaling pathways activated by IL-6 and ligands of CXCR2.

## Results

### Elimination of senescent cells impairs liver regeneration.

**Figure 1:**
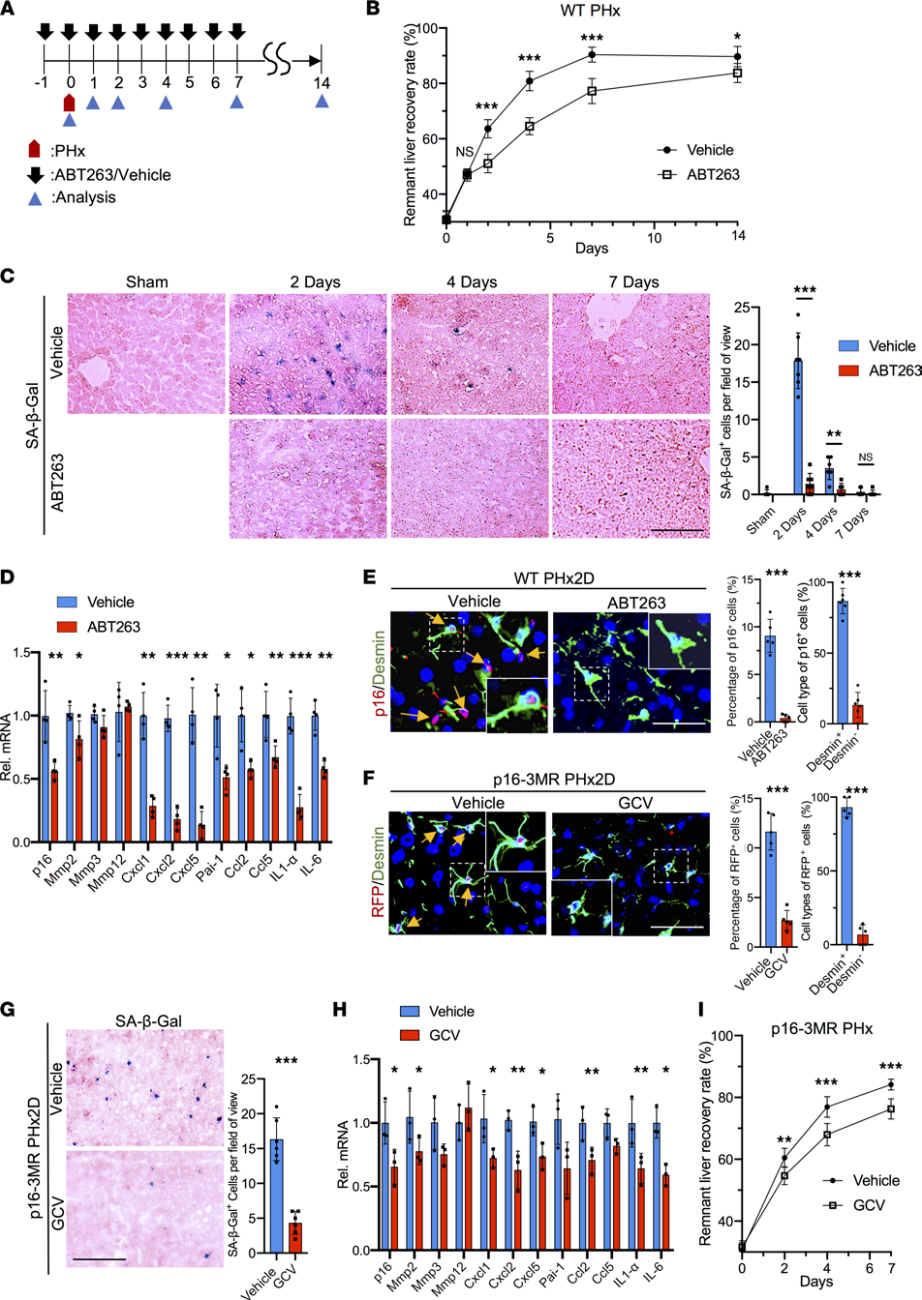
Elimination of senescent cells impairs liver regeneration. (**A**) Scheme showing mice subjected to PHx and analyzed thereafter. ABT263 (100 mg/kg/d) or vehicle (DMSO) was injected daily 1 day before PHx for 15 days. (**B**) Remnant liver recovery rates are shown (*n* = 3 at days 0, 1, and 14; *n* = 8 day 2; *n* = 5 day 4; *n* = 4 day 7). (**C**) Frozen liver sections were stained for SA-β-Gal activity (blue) and counterstained with eosin (pink). SA-β-Gal^+^ cells per microscopic field were quantified (sham *n* = 3; day 2, *n* = 7; days 4 and 7, *n* = 6). (**D**) Liver expression of the SASP 2 days post-PHx was quantified by quantitative reverse transcription PCR (qRT-PCR) (*n* = 4). (**E**) Liver sections from vehicle- or ABT263-treated WT mice 2 days post-PHx (PHx2D) were immunostained for p16 (red) and desmin (green) and counterstained with DAPI (blue). Arrows point to p16^+^ cells (*n* = 5). Percentage of p16^+^ cells that were desmin^+^ or desmin^–^ was quantified by cell counting (*n* = 6). (**F**) p16-3MR mice were treated daily with GCV or vehicle (PBS) after PHx. Liver sections 2 days post-PHx were immunostained for RFP (red) and desmin (green) and counterstained with DAPI (blue). Arrows point to RFP^+^ cells, quantified by cell counting (*n* = 5). (**G**) The frozen liver sections were stained for SA-β-Gal activity (blue) and counterstained with eosin (pink). SA-β-Gal^+^ cells (*n* = 6) were counted. (**H**) Expression of *p16* and genes of the SASP in the liver 2 days post-PHx was quantified by quantitative PCR (*n* = 3). (**I**) The remnant liver recovery rates of vehicle- and GCV-treated p16-3MR mice after PHx (*n* = 3 day 0, *n* = 6 day 2, *n* = 4 days 4 and 7; *P* < 0.033 by 2-way ANOVA for comparison of the remnant liver recovery curves). Scale bars: 50 μm. Data expressed as mean ± SD. *P < 0.033, **P < 0.002, ***P < 0.001 by Student’s *t* test (**C**–**H**) or 2-way ANOVA (**B** and **I**).

To assess whether senescent cells contribute to liver regeneration after PHx, we used the senolytic drug ABT263 (Navitoclax), which blocks the antiapoptotic proteins Bcl-2 and Bcl-xL, to induce apoptosis in senescent cells (25, 26). Young (2–3 months old) wild-type (WT) male C57BL/6 mice were injected with ABT263 1 day before PHx and daily thereafter (Figure 1A). Remarkably, the remnant liver recovery rate of ABT263-treated mice was significantly lower than that of vehicle-treated controls from 2–14 days after PHx, with a reduction from 64% to 52% on day 2 postsurgery (Figure 1B). Both vehicle- and ABT263-treated mice showed similar liver tissue morphology and comparable liver damage as judged by serum levels of alanine aminotransferase (ALT) and aspartate transaminase (AST), neutrophil infiltration measured by myeloperoxidase (MPO) activity and Ly6G staining, and macrophage numbers assessed by F4/80 staining (Supplemental Figure 1, A–D; supplemental material available online with this article; https://doi.org/10.1172/jci.insight.158207DS1). Senescent cells positive for senescence-associated β-galactosidase (SA-β-Gal) appeared in large numbers in vehicle-treated control mice 2 days after PHx and declined thereafter, becoming undetectable by day 7 (Figure 1C). SA-β-Gal^+^ cells were essentially absent in the livers of ABT263-treated mice, consistent with the loss of senescent cells. Moreover, expression of the senescence marker gene *p16^INK4a^* and of genes characteristic of the SASP was greatly reduced in ABT263-treated mice 2 days after PHx, when a large reduction in SA-β-Gal^+^ cells was observed (Figure 1D). The majority (87%) of p16^+^ senescent cells stained positive for desmin, a marker for HSCs (27), indicating that they were derived from HSCs (Figure 1E). By contrast, only 7% of p16^+^ senescent cells stained positive for albumin, a marker for hepatocytes (Supplemental Figure 1E) (28). Desmin^+^ apoptotic cells appeared in ABT263-treated mice but not in control mice as judged by costaining for cleaved caspase-3 (Supplemental Figure 1F). Furthermore, primary HSCs isolated from WT mice 2 days after PHx showed increased SA-β-Gal^+^ and p16^+^ senescent cells and elevated expression of SASP, but HSCs from sham-operated or ABT263-treated mice did not (Supplemental Figure 2, A–C). These results showed that HSC-derived senescent cells quickly accumulated in regenerating livers within 2 days after PHx, and ABT263 effectively eliminated them by inducing apoptosis.

To exclude the possibility that defects in liver regeneration might be due to off-target effects of ABT263, we sought to eliminate senescent cells using a different approach. The p16-3MR transgenic mouse expresses Renilla luciferase (LUC), red fluorescent protein (RFP), and herpes simplex virus thymidine kinase (HSV-TK) under the promoter of *p16^INK4a^*, a gene whose expression is linked to senescence (17, 29). These mice allow the detection and sorting of senescent cells by LUC and RFP, and the killing of these cells by ganciclovir (GCV), a nucleoside analog that is converted by HSV-TK into a toxic DNA chain terminator (30). Thus, we treated p16-3MR mice with GCV (30 mg/kg/d) daily after PHx. As expected, GCV-treated p16-3MR mice exhibited greatly reduced SA-β-Gal^+^ and RFP^+^desmin^+^ senescent cells after PHx (Figure 1, F and G), and the expression of *p16^INK4a^* and SASP was also diminished (Figure 1H). Importantly, p16-3MR mice treated with GCV also showed impaired liver weight recovery after PHx compared with vehicle-treated controls (Figure 1I), supporting the notion that senescent cells play important roles in liver regeneration.

Following PHx, most new hepatocytes are derived from preexisting hepatocytes that reenter the cell cycle from the quiescent G_0_ phase (31–33). The peak of hepatocyte proliferation occurred 2 days post-PHx, concomitant with the accumulation of senescent cells (Supplemental Figure 3, A–C). Livers of both ABT263-treated WT and GCV-treated p16-3MR mice showed markedly fewer Ki67^+^ proliferating hepatocytes 2 days post-PHx in zone 1 (periportal) and zone 2 (midlobular), whereas no significant difference was observed in zone 3 (pericentral) (Supplemental Figure 3, A–C). This finding is consistent with previous observations that hepatocyte proliferation after PHx progresses from zone 1 to zone 2 to zone 3, with most proliferation occurring in zones 1 and 2 during the first 72 hours (2, 33). Liver regeneration was largely complete by 7–14 days in control mice, and Ki67^+^ hepatocytes became undetectable, whereas hepatocyte proliferation in mice with senescent cell elimination continued at low levels as the liver mass was not fully restored.

### Senescent HSCs promote hepatocyte proliferation through the SASP.

**Figure 2:**
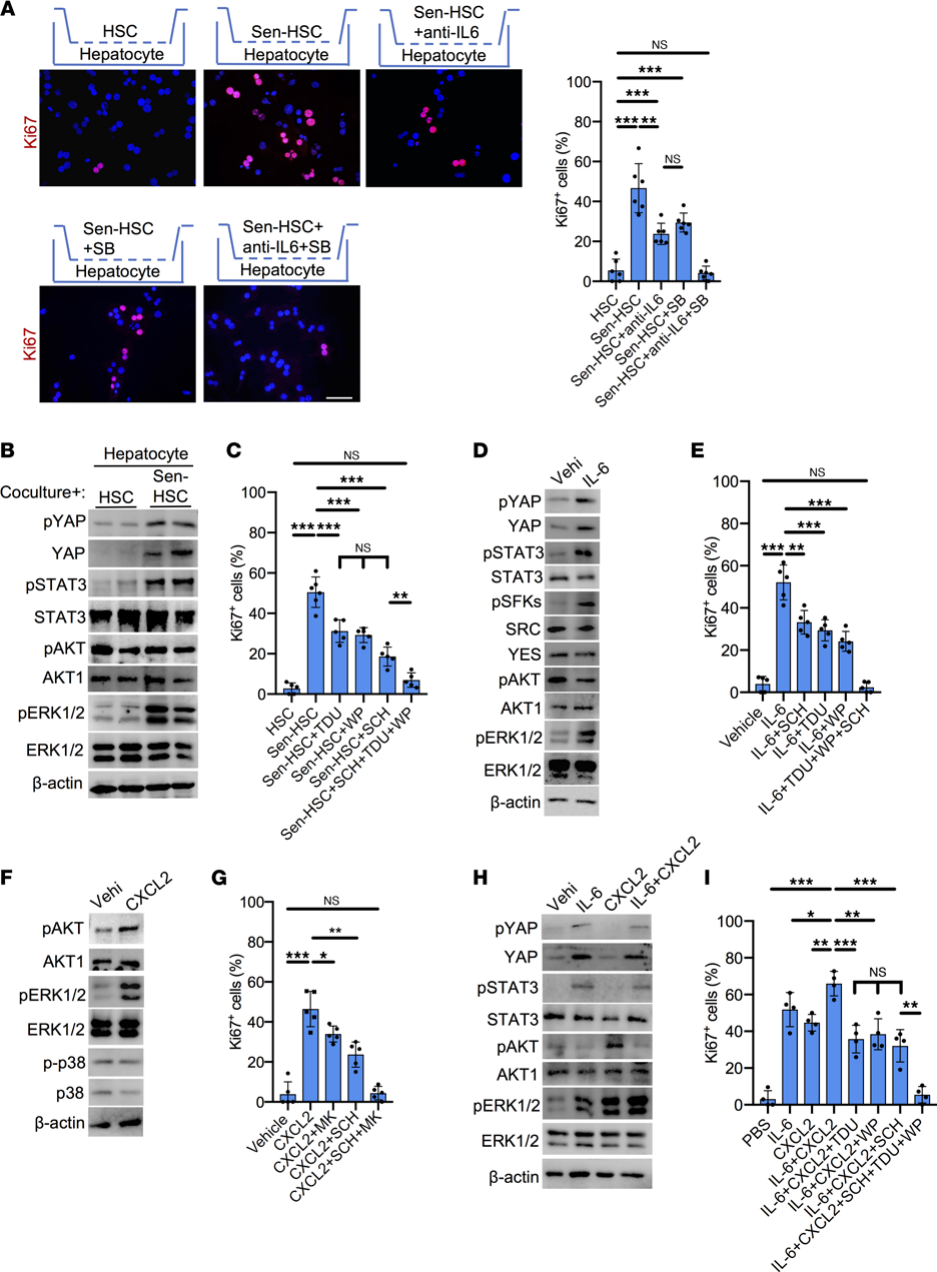
SASP of senescent HSCs induces hepatocyte proliferation. (**A**) Primary hepatocytes isolated from WT male livers were cocultured with HSCs, senescent HSCs (Sen-HSC), and Sen-HSC with anti–IL-6 antibody (5 μg/mL) and/or SB225002 (SB; 1 μM) as indicated for 24 hours, then stained with anti-Ki67 (red). Ki67^+^ cells were quantified by counting (*n* = 6). (**B**) Immunoblots of protein extracts from hepatocytes cocultured with HSCs or Sen-HSCs were probed with antibodies against phosphorylated (p-) YAP (Y357), YAP, p-STAT3 (Y705), STAT3, p-AKT (S473), AKT1, p-ERK1/2 (T202/Y204), ERK1/2, and β-actin. (**C**) Primary hepatocytes cocultured with HSCs, Sen-HSC, and Sen-HSC with Super-TDU (TDU; 50 nM), WP1066 (WP; 5 μM), and SCH772984 (SCH; 1 μM) as indicated for 24 hours and were stained with anti-Ki67 antibodies. Ki67^+^ cells were counted (*n* > 5). (**D**) Immunoblots of protein extracts from hepatocytes treated with vehicle (PBS) or IL-6 (10 ng/mL) for 24 hours were probed with indicated antibodies. (**E**) Hepatocytes treated with vehicle, IL-6, and IL-6 with SCH, TDU, and WP as indicated for 24 hours were stained with Ki67 antibodies and Ki67^+^ cells counted (*n* = 5). (**F**) Immunoblots of protein extracts from hepatocytes treated with vehicle or CXCL2 (10 ng/mL) for 24 hours were probed with indicated antibodies and antibodies against p-p38 (T180/Y182) and p38. (**G**) Hepatocytes were treated with vehicle, CXCL2, MK2206 (MK; 1 μM), SCH, or in combination as indicated for 24 hours and were stained with Ki67 antibodies. Ki67^+^ cells were counted (*n* = 5). (**H**) Immunoblots of protein extracts from vehicle-, IL-6-, CXCL2-, or IL-6+CXCL2-treated hepatocytes for 24 hours were probed with indicated antibodies. (**I**) Hepatocytes treated with PBS, IL-6, CXCL2, IL-6+CXCL2, or IL-6+CXCL2 together with SCH, TDU, and WP alone and in combination for 24 hours were stained with anti-Ki67 antibodies; Ki67^+^ cells were counted (*n* = 4). Scale bar: 50 μm. Data expressed as mean ± SD. *P < 0.033, **P < 0.002, ***P < 0.001, Student’s *t* test.

Senescent cells exert effects on neighboring cells through the expression of SASP (18). Our results showed increased expression of the SASP in senescent HSCs (Supplemental Figure 2C), consistent with previously characterized alterations in the transcriptome and secretome of senescent HSCs (34, 35). To test whether liver regeneration may be stimulated by the SASP, we first examined the paracrine effects of senescent HSCs on hepatocyte proliferation using a Transwell coculture system. Freshly isolated hepatocytes were seeded in the bottom chamber, then cocultured with primary HSCs that were either treated with vehicle or rendered senescent by exposure to mitomycin-C (MMC) placed in the top chamber (36, 37). Cells in the top and bottom chambers did not contact each other but shared a common growth medium, allowing paracrine action of secreted molecules. Cellular senescence in HSCs induced by MMC was confirmed by proliferation arrest, morphological changes, SA-β-Gal staining, and p16 and SASP expression (Supplemental Figure 4, A–C). Hepatocytes cocultured with MMC-treated senescent HSCs, but not vehicle-treated HSCs, showed approximately 10-fold increase in the number of Ki67^+^ proliferating cells (Figure 2A), indicating that senescent HSCs can substantially stimulate hepatocyte proliferation in a paracrine manner.

Among the cytokines of the hepatic SASP (Figure 1, D and H), IL-6 is known to regulate hepatocyte proliferation, and *IL-6*–knockout mice have impaired liver regeneration (38, 39). Indeed, the inclusion of monoclonal anti–IL-6 antibody (5 μg/mL) in the coculture inhibited senescent HSC-stimulated hepatocyte proliferation by approximately 50% (Figure 2A), indicating that IL-6 secreted by senescent HSCs plays an important role in stimulating hepatocyte proliferation. In addition to IL-6, the hepatic SASP also included the expression of ligands of the chemokine receptor CXCR2, including the CXC chemokines CXCL1 (KC), CXCL2 (MIP2), and CXCL5 (ENA78; Figure 1, D and H). CXC chemokines have proliferative effects on hepatocytes, and CXCR2-knockout mice experience delayed liver regeneration after PHx (40, 41). Inclusion of the CXCR2 antagonist SB225002 in the coculture curtailed senescent HSC-stimulated hepatocyte proliferation by approximately 40% (Figure 2A), indicating that CXCR2 signaling also plays an important role. Remarkably, the combination of anti–IL-6 antibody and SB225002 reduced hepatocyte proliferation to background level in the coculture (Figure 2A). To further assess the role of IL-6 and CXCR2 in senescent HSC-stimulated hepatocyte proliferation, we used siRNA to knock down *Il-6* and *Cxcr2* expression. Knockdown of either *Il-6* in HSCs or *Cxcr2* in hepatocytes partially reduced senescent cell–stimulated hepatocyte proliferation, whereas knockdown of both *Il-6* and *Cxcr2* completely abrogated senescent cell–dependent hepatocyte proliferation in the coculture (Supplemental Figure 5, A and B). Together, these results showed that IL-6 and CXCR2 ligands are the essential components of the SASP that stimulated hepatocyte proliferation.

### SASP-dependent activation of IL-6 receptor/glycoprotein 130 and CXCR2 signaling pathways.

IL-6 interacts with its coreceptors IL-6R and glycoprotein 130 (gp130) to regulate multiple signaling pathways, including JAK/STAT3, SRC family kinase/Yes-associated protein (SFK/YAP), Src homology phosphotyrosyl phosphatase 2/RAS/ERK, and PI3K/AKT/mTOR (42, 43). IL-6 engagement of IL-6R and gp130 leads to the recruitment of JAK and STAT3, whereupon STAT3 is phosphorylated by JAKs at Y705, leading to STAT3 dimerization, nuclear translocation, and function as a transcriptional activator (44). SFKs include SRC and Yes, which can activate the transcriptional regulator YAP by phosphorylation at Y357 to promote its stability and nuclear translocation (45). Consistent with the interpretation that IL-6 contributes to SASP-induced hepatocyte proliferation, senescent HSCs stimulated the activation of STAT3 (Y705) and YAP (Y357) by phosphorylation in hepatocytes (Figure 2B). Phosphorylation of ERK1/2 at T202/Y204 was also greatly increased, though this can be due to both IL-6R/gp130 and CXCR2 signaling. Indeed, SASP-induced hepatocyte proliferation was partially blocked by inhibitors of YAP (Super-TDU), STAT3 (WP1066), or ERK1/2 (SCH772984; Figure 2C). As expected, the abilities of WP1066 to inhibit STAT3 phosphorylation, SCH772984 to block ERK1/2 activation, and Super-TDU to curtail the expression of YAP target genes were observed in hepatocytes, showing the efficacy of these inhibitors (Supplemental Figure 5, C–E). Remarkably, combined inhibition of YAP, STAT3, and ERK1/2 essentially abrogated SASP-induced hepatocyte proliferation, indicating that these signaling pathways account for senescent cell–stimulated hepatocyte proliferation through the SASP.

To further analyze the SASP-associated signaling pathways for hepatocyte proliferation, we stimulated primary hepatocytes with recombinant IL-6 and CXCL2, a CXCR2 ligand. As expected, hepatocytes treated with IL-6 alone showed activation of SFKs (Y416) (46, 47), YAP (Y357), and ERK1/2 (T202/Y204; Figure 2D). Although IL-6 can induce the activation of AKT in some cancer cells, phosphorylation of AKT is inhibited by IL-6 in hepatocytes (48, 49). The addition of IL-6 to hepatocytes enhanced their proliferation by >12-fold, which was substantially reduced by the YAP inhibitor Super-TDU (~42%), the STAT3 inhibitor WP1066 (~46%), or the ERK inhibitor SCH772984 (~35%) (Figure 2E). Combined inhibition of YAP, STAT3, and ERK1/2 completely abrogated the IL-6–stimulatory effects on hepatocyte proliferation, indicating that these are the critical pathways mediating IL-6 functions.

CXCR2 is a G protein–coupled receptor that upon ligand binding activates multiple downstream G protein signaling cascades, including RAS/ERK, PI3K/AKT, phospholipase C/PKC, and MAPK/p38, some of which are linked to its chemotactic activity (50). The addition of recombinant CXCL2 (10 ng/mL) to primary hepatocytes strongly stimulated hepatocyte proliferation by >10-fold with robust activation of ERK1/2 (T202/Y204) and AKT (S473) but not activation of p38 (T180/Y182; Figure 2, F and G). Inhibition of AKT by MK2206 or ERK1/2 by SCH77298 partially curtailed CXCL2-induced hepatocyte proliferation, whereas inhibition of both AKT and ERK1/2 completely abrogated the CXCL2 effect (Figure 2G). Thus, CXCL2 stimulates hepatocyte proliferation primarily through AKT and ERK1/2.

Remarkably, when both IL-6 and CXCL2 were added to hepatocytes in combination, the activation of ERK1/2 was further elevated, and hepatocyte proliferation was enhanced above the level stimulated by either protein alone (Figure 2, H and I). Similar to senescent cell–induced paracrine action (Figure 2, B and C), hepatocyte proliferation induced by IL-6 and CXCL2 together was partially blocked by inhibition of YAP (Super-TDU), STAT3 (WP1066), or ERK1/2 (SCH772984; Figure 2I), and the combined inhibition of YAP, STAT3, and ERK1/2 essentially abrogated hepatocyte proliferation. As expected, the activation of AKT by CXCL2 was suppressed by the presence of IL-6 (Figure 2H) (48, 49), and consistently, we observed no activation of AKT in hepatocytes stimulated by senescent HSCs (Figure 2B). Together, these results show that senescent HSCs strongly stimulate hepatocyte proliferation in a coculture system through IL-6 and CXCR2 ligands secreted as part of the SASP, which act through a combination of YAP, STAT3, and enhanced activation of ERK1/2 (Figure 2, B and H).

### Activation of IL-6R/gp130 and CXCR2 signaling in response to PHx.

**Figure 3:**
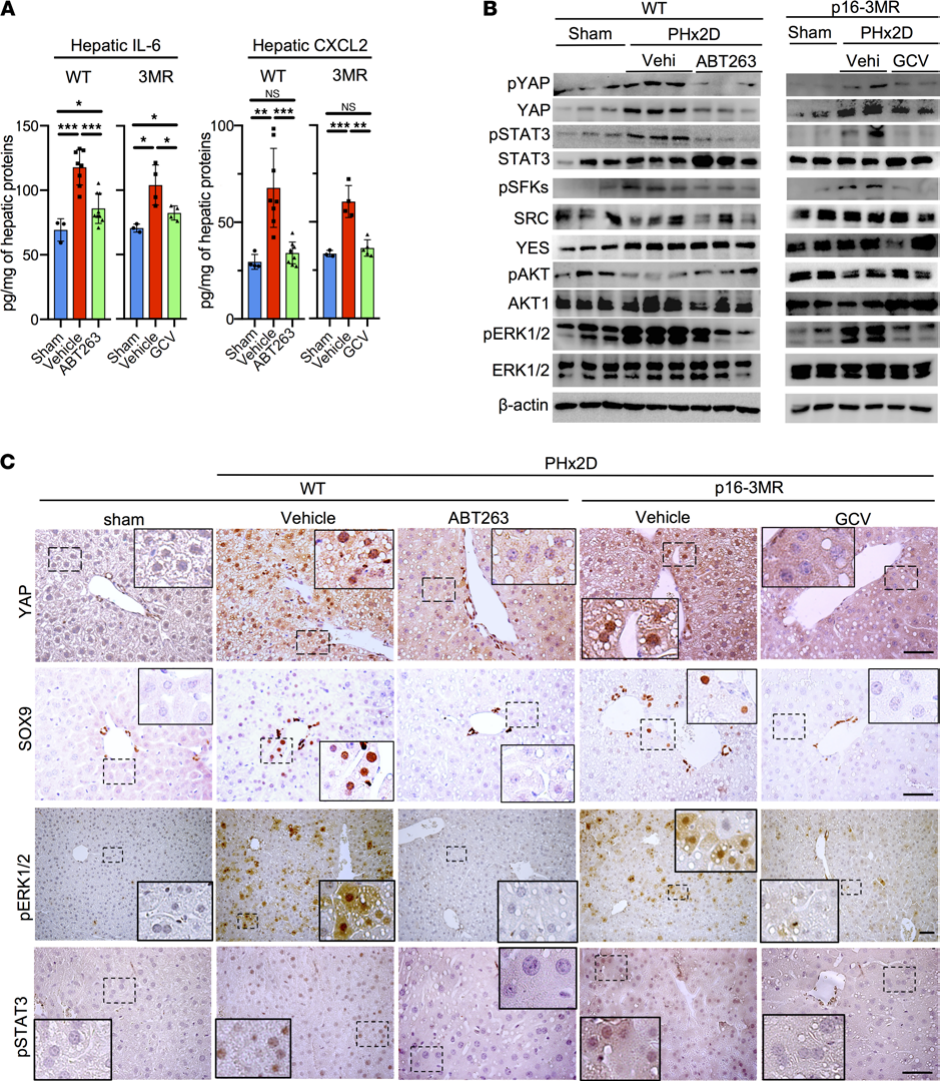
Senescent cells stimulate YAP, STAT3, and ERK1/2 activation after PHx. (**A**) The IL-6 and CXCL2 protein levels in whole liver lysates were quantified by ELISA, including lysates from sham-operated (*n* = 3) WT and p16-3MR (*n* = 4) mice, vehicle- or ABT263-treated WT mice 2 days after PHx (*n* = 8 each), and vehicle- or GCV-treated p16-3MR mice 2 days after PHx (*n* = 4 each). (**B**) Immunoblots of protein extracts from the indicated livers 2 days after PHx (PHx2D) were probed with the indicated antibodies. (**C**) Liver sections of sham-operated, vehicle- and ABT263-treated WT mice, or vehicle- and GCV-treated p16-3MR mice 2 days after PHx were stained with anti-YAP, anti-SOX9, anti–p-ERK1/2, or anti–p-STAT3 antibodies and counterstained with hematoxylin. Scale bars: 50 μm. Data expressed as mean ± SD. *P < 0.033, **P < 0.002, ***P < 0.001, Student’s *t* test. SOX9, SRY-box transcription factor 9.

Since IL-6 and CXCR2 ligands secreted by senescent HSCs are responsible for stimulating hepatocyte proliferation in vitro (Figure 2A), we sought to assess their senescent cell–dependent expression and function in vivo. The hepatic levels of both IL-6 and CXCL2 were substantially increased 2 days after PHx as measured by ELISA (Figure 3A). However, their increases were largely eliminated in mice with the removal of senescent cells by ABT263 or GCV, confirming that senescent cells are major sources of their expression after PHx (Figure 3A). Activation of signaling molecules downstream of IL-6R/gp130 and CXCR2 by phosphorylation including STAT3, SFKs, YAP, and ERK1/2 was evident in WT or p16-3MR mice after PHx, but phosphorylation was substantially reduced with the removal of senescent cells by ABT263 in WT mice or GCV in p16-3MR mice (Figure 3B). Immunohistochemistry also showed activation of YAP, ERK1/2, and STAT3 in the liver concomitant with hepatocyte proliferation after PHx, but activation of these molecules was abolished with the removal of senescent cells by ABT263 or GCV (Figure 3C). YAP activation in liver regeneration has been shown to reprogram a subset of periportal hepatocytes into SOX9^+^ progenitor-like cells (PLCs) that can give rise to both cholangiocytes and hepatocytes (51, 52). Although these SOX9^+^ PLCs contribute only modestly to the repopulation of hepatocytes in PHx, these bipotential cells may provide an alternative mechanism for regeneration should the proliferation of existing hepatocytes be blunted (53, 54). SOX9^+^ hepatocytes were increased along with elevated nuclear YAP after PHx, but hepatocytes with nuclear YAP and SOX9 were not detected in the livers of mice with senescent cell removal (Figure 3C). Likewise, the expression of YAP target genes was also diminished with the elimination of senescent cells (Supplemental Figure 6A). However, *Yap* mRNA level was unchanged, suggesting that *Yap* is regulated on the posttranscriptional level.

IL-6 is known to activate SFKs, which in turn activates YAP by tyrosyl phosphorylation at Y357 (Figure 2D and Figure 3B) (45). Mice with liver-specific deletion of *Yap* or *Yap* and *Taz* suffer significantly blunted liver weight restoration after PHx (55, 56). Thus, we used PP2 (57), an SFK inhibitor, to probe the involvement of SFKs in YAP activation in liver regeneration. Mice injected with PP2 had reduced liver weight restoration from 65% to 60% at 2 days post-PHx (Supplemental Figure 6, B and C). Activation of SFKs (Y416) in the liver was abrogated, with a concomitant reduction in total YAP protein level, p-YAP (Y357), and Ki67^+^ cells (Supplemental Figure 6, D and E). These findings show that PP2-inhibitable SFKs are critical for the activation of YAP, which promotes hepatocyte proliferation after PHx (55, 56).

We showed above that IL-6 and CXCL2 together strongly activated ERK1/2. To further assess the role of ERK, we injected the ERK inhibitor SCH772984 daily into WT mice. This resulted in inhibition of ERK1/2 phosphorylation, reduced number of Ki67^+^ proliferating cells, and impaired liver weight restoration from 63% to 54% 2 days after PHx (Supplemental Figure 7, A–D). These results show that ERK activation plays an important role in liver regeneration after PHx, though ERK1/2 is also thought to mediate the activity of other growth factors important for liver regeneration such as HGF and is thus not exclusive to senescence-driven regeneration (58). However, the expression of *c-fos*, an ERK-regulated gene that can promote hepatocyte cell cycle reentry through the regulation of cyclin D1 (59, 60), was elevated after PHx but eliminated by ABT263 or SCH772984, indicating that *c-fos* expression after PHx is dependent on the presence of senescent cells and ERK activation (Supplemental Figure 7E).

### IL-6 and CXCL2 fully restore liver regeneration in ABT263-treated mice.

**Figure 4:**
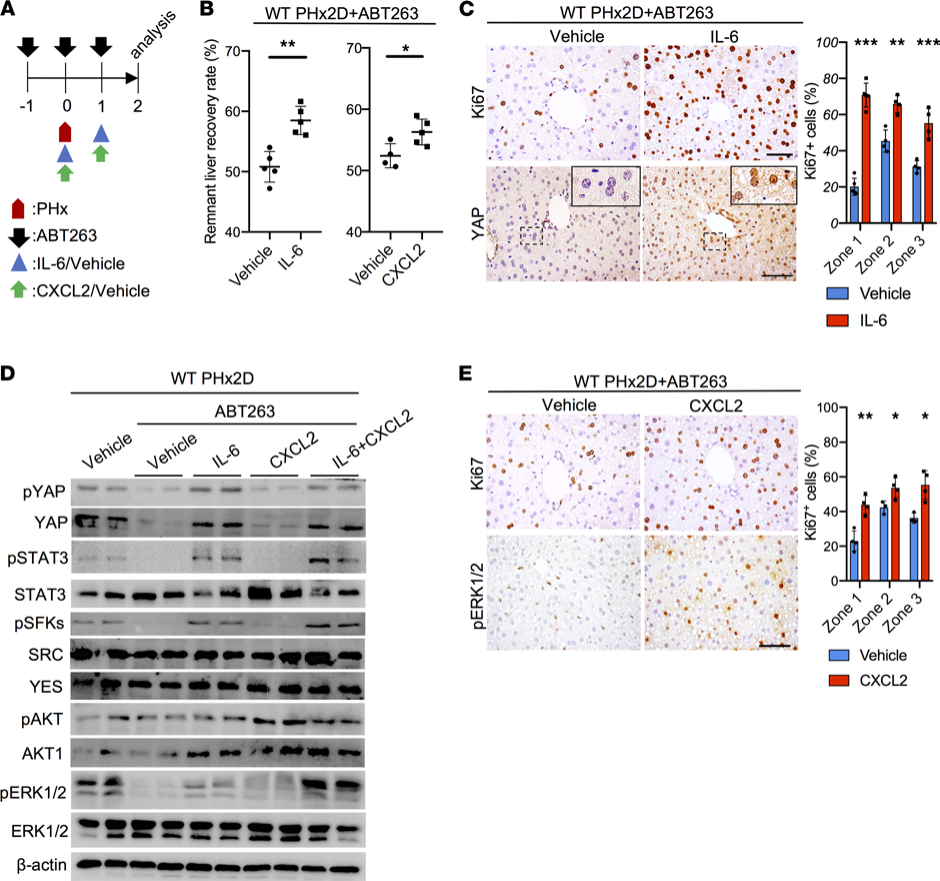
IL-6 or CXCL2 promotes liver regeneration in ABT263-treated mice. (**A**) Experimental scheme of ABT263-treated WT male mice subjected to PHx with vehicle (PBS), IL-6 (50 μg/kg/d), or CXCL2 (15 μg/kg/d) injections. (**B**) The remnant liver recovery rates of the indicated livers were measured 2 days after PHx (PHx2D; *n* = 4–5). (**C**) Liver sections (zone 1) of WT mice subjected to ABT263 and PHx followed by injections of vehicle or IL-6 were stained with anti-Ki67 or anti-YAP antibodies 2 days after PHx. The percentages of Ki67^+^ hepatocytes in all 3 zones were quantified by cell counting (*n* = 4). (**D**) Immunoblots of protein extracts from whole liver lysates of indicated mice were probed with the indicated antibodies. ABT263-treated mice were injected with vehicle, IL-6, CXCL2, or both on days 0 and 1 after PHx, then harvested on day 2 for analysis. (**E**) Liver sections of WT mice subjected to ABT263 and PHx followed by injections of vehicle or CXCL2 were stained with anti-Ki67 (zone 1) or anti–p-ERK1/2 antibodies 2 days after PHx. The percentages of Ki67^+^ hepatocytes in all 3 zones were quantified by cell counting (*n* = 4). Scale bar: 50 μm. Data are expressed as mean ± SD. *P < 0.033, **P < 0.002, ***P < 0.001, Student’s *t* test.

To test the hypothesis that senescent cells promote liver regeneration through IL-6 and CXCR2 ligands, we first injected recombinant IL-6 (50 μg/kg, i.p.) daily into ABT263-treated mice after PHx (Figure 4A). Indeed, liver weight recovery was markedly improved after IL-6 treatment 2 days post-PHx, increasing from 51% to 58% (Figure 4B). Hepatocyte proliferation was preferentially enhanced in zone 1, suggesting that these hepatocytes are most responsive to IL-6 (Figure 4C). ABT263-treated mice after PHx sustained impaired phosphorylation of the signaling proteins downstream of IL-6R/gp130, including STAT3, SFKs, and YAP, but their phosphorylation was restored by the administration of IL-6 (Figure 4D). There was also a modest enhancement of ERK1/2 activation. Total and nuclear YAP levels were elevated in mice treated with IL-6 (Figure 4, C and D). These results show that whereas IL-6 protein and signaling were diminished in mice with senescent cell elimination, injection of IL-6 substantially restored YAP and STAT3 activation, hepatocyte proliferation, and liver regeneration after PHx.

Next, we tested whether the administration of a CXCR2 ligand can restore liver regeneration in mice deficient in senescent cells. Injection of recombinant CXCL2 (15 μg/kg/d) into ABT263-treated WT mice after PHx enhanced liver weight restoration from 52% to 56% and increased Ki67^+^ hepatocytes 2 days after PHx (Figure 4, A, B, and E). Whereas ABT263 treatment reduced the activation of signaling molecules downstream of IL-6 and CXCR2 ligands (Figure 4D), injection of CXCL2 partially restored the activation of ERK1/2 and *c-fos* expression without affecting the activation of YAP or STAT3 (Figure 4D and Supplemental Figure 7E).

**Figure 5:**
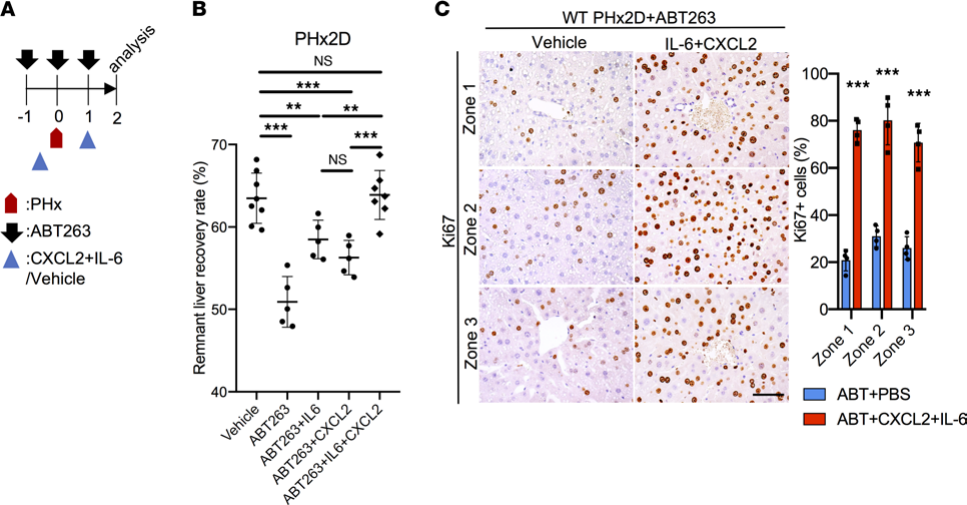
IL-6 and CXCL2 restore liver recovery rate in mice with senescent cell elimination. (**A**) Experimental scheme of ABT263-treated WT male mice subjected to PHx, followed by injections of vehicle (PBS) or a combination of IL-6 (50 μg/kg/d) and CXCL2 (15 μg/kg/d). (**B**) The remnant liver recovery rates were assessed 2 days after PHx. Injection of IL-6 and CXCL2 fully restored liver mass recovery to the level of WT mice without ABT263 exposure (ABT263+IL-6+CXCL2, *n* = 7; ABT263 control, *n* = 5). Data on mice subjected to PHx treated with vehicle, ABT263+IL-6, and ABT263+CXCL2 were drawn from Figure 1B and Figure 4B. (**C**) Liver sections of indicated mice were stained with anti-Ki67 antibodies to reveal proliferating cells in all 3 zones, and the numbers of Ki67^+^ cells were counted (*n* = 4). Scale bar: 50 μm. Data expressed as mean ± SD. **P < 0.002, ***P < 0.001, Student’s *t* test.

To test the combined effects of IL-6 and CXCL2, we injected both into WT mice subjected to PHx after senescent cell elimination by ABT263 (Figure 5A). In hepatocytes, IL-6 specifically enhanced YAP and STAT3 activation, and CXCL2 induced AKT activation (Figure 2, D and F). Interestingly, the combinatorial administration of IL-6 and CXCL2 in mice induced a robust synergistic ERK1/2 activation, but AKT activation was effectively repressed, similar to the effects of SASP in cultured hepatocytes (Figure 2B and Figure 4D). Remarkably, the combination of IL-6 and CXCL2 dramatically boosted the remnant liver recovery rate 2 days after PHx, concomitant with a large increase in Ki67^+^ proliferating hepatocytes in all 3 zones (Figure 5, B and C). Whereas treatment with IL-6 and CXCL2 increased remnant liver recovery rate from approximately 51% to 58% and 56%, respectively, the combination of both fully restored the liver recovery rate to 64%, the same level as mice without senescent cell elimination (Figure 5B). Collectively, these results show that senescent HSCs stimulate liver regeneration by the SASP mediated through the combined effects of IL-6 and CXCR2 ligands.

### CCN1 induces cellular senescence and contributes to liver regeneration after PHx.

**Figure 6:**
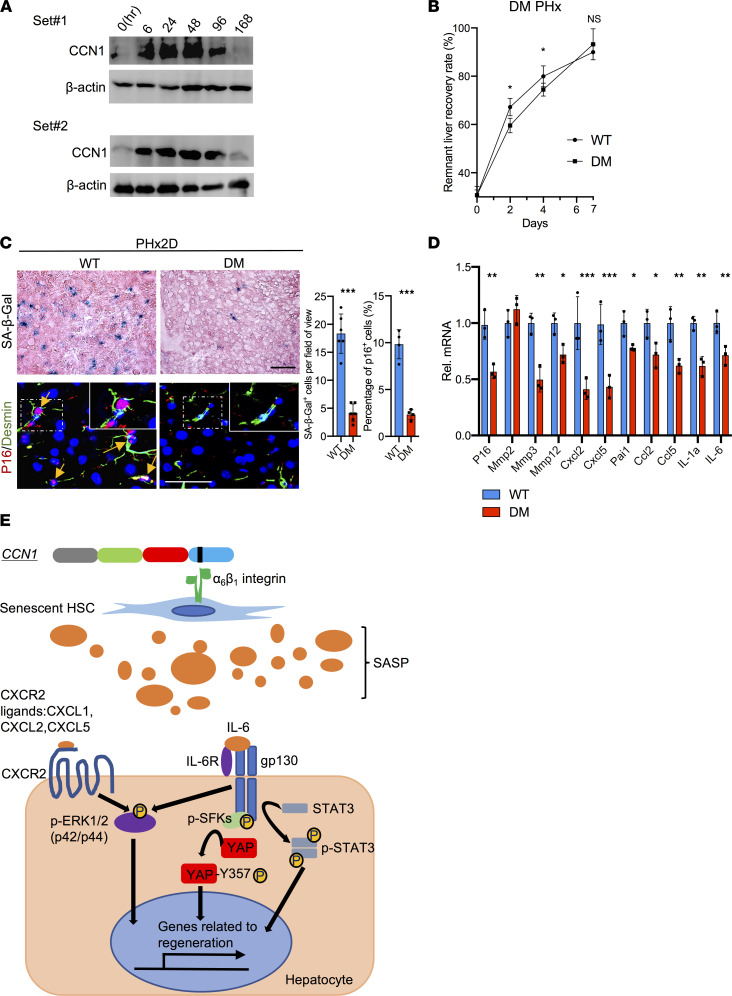
CCN1 induces senescence in HSCs and contributes to liver regeneration. (**A**) Liver protein extracts from WT mice at the indicated times (hours) after PHx were immunoblotted and probed with anti-CCN1 and anti–β-actin antibodies. (**B**) The remnant liver recovery rates were assessed for WT and *Ccn1^DM/DM^* (DM) mice at indicated times after PHx (day 0, *n* = 3; day 2 and 7, *n* = 4; day 4, *n* = 8). (**C**) SA-β-Gal staining of frozen liver sections from WT and DM mice 2 days after PHx. Liver sections were also double immunostained for p16 (red) and desmin (green) and counterstained with DAPI (blue). Yellow arrowheads are pointing to p16^+^ cells. The number of SA-β-Gal^+^ cells (*n* = 6) per microscopic field and percentage of p16^+^ cells (*n* = 4) were quantified by cell counting. (**D**) Expression of genes related to cell cycle arrest and the SASP in WT and *Ccn1^DM/DM^* mouse livers 2 days after PHx was measured by qRT-PCR (*n* = 3). (**E**) Schematic diagram illustrating proposed SASP signaling pathways contributing to liver regeneration. The matricellular protein CCN1 is released upon injury and induces cellular senescence in HSCs through integrin α_6_β_1_ within 2 days after PHx. Senescent cells secrete proteins of the SASP critical to liver regeneration, including IL-6, which engages IL-6R and gp130 to induce hepatocyte proliferation through activation of STAT3 and SFK-mediated phosphorylation of YAP. IL-6 also synergizes with CXCR2 ligands secreted as part of the SASP, including CXCL1, CXCL2, and CXCL5, to induce a robust activation of ERK1/2 that promotes hepatocyte proliferation. Scale bars: 50 μm. Data expressed as mean ± SD. *P < 0.033, **P < 0.002, ***P < 0.001 assessed by Student’s *t* test (**C** and **D**) or 2-way ANOVA (**B**).

Although senescent cells are known to emerge during aging from accumulated cellular stress and DNA damage (61), specific factors such as TGF-β can also induce senescence in a context-dependent manner (19, 20). We have previously shown that the matricellular protein CCN1 can induce cellular senescence in activated HSCs through direct binding to integrin α_6_β_1_, thereby accelerating matrix remodeling and resolution of liver fibrosis induced by CCl_4_ or cholestasis (22). CCN1 is released from activated platelets and macrophages (62, 63) and thus can mediate signals from injuries and hemostasis. Indeed, the hepatic CCN1 protein level was quickly elevated within 6 hours post-PHx and sustained for 96 hours, declining to basal level by 168 hours as the liver weight was restored (Figure 6A). To assess the potential role of CCN1-induced senescence, we performed PHx in *Ccn1^DM/DM^* knockin mice, which encode a mutant CCN1 that is unable to bind integrin α_6_β_1_ and therefore defective for the induction of senescence (16, 22). Remarkably, *Ccn1^DM/DM^* mice experienced impaired liver weight restoration on days 2 and 4 post-PHx compared with WT mice, though regeneration recovered by day 7 (Figure 6B). Importantly, *Ccn1^DM/DM^* livers showed very few SA-β-Gal^+^ or p16^+^desmin^+^ senescent cells, and expression of SASP was attenuated, confirming that CCN1 is a key inducer of HSC senescence following PHx (Figure 6, C and D). *Ccn1^DM/DM^* mice also showed reduced hepatocyte proliferation, fewer YAP^+^ and SOX9^+^ hepatocytes, decreased expression of YAP target genes, and curtailed activation of IL-6R/gp130 downstream effectors (Supplemental Figure 8, A–D). Thus, *Ccn1^DM/DM^* livers failed to develop senescent HSCs, leading to reduced SASP signaling and hepatocyte proliferation, similar to mice with senescent cell elimination by ABT263. These results show that CCN1 is rapidly induced after PHx and stimulates senescence in HSCs, which express the SASP to promote hepatocyte proliferation and liver regeneration.

## Discussion

The accumulation of senescent cells can profoundly influence the aging process, the efficacy of tissue repair, and the pathology of diseases including cancer (12, 64). By exerting diverse effects on their cellular microenvironment, senescent cells can evoke either beneficial or detrimental biological consequences in a context-dependent manner. For example, cellular senescence can be tumor suppressive by preventing damaged cells from becoming oncogenic, but the SASP secreted by senescent cells can also promote angiogenesis and stimulate the proliferation and migration of tumor cells, thereby enhancing malignancy (65). Both the senescent cell type and how the cell becomes senescent may influence the SASP and the biological outcome. The accumulation of senescent parenchymal cells will likely compromise tissue regeneration by impeding their proliferation, whereas senescence in supportive cells may play different roles. A general observation is that senescence occurring through chronic injury may be detrimental, whereas acutely induced senescence is more likely to be beneficial (66).

Age-related accumulation of macromolecular damage can cause cells to suffer a broad range of defects and dysfunctions, some of which may manifest as aspects of the senescence phenotype. A recent study found that 6- to 8-month-old adult mice showed age-related hepatic dysfunctions including pronounced vacuolization, lipidosis, and impaired regeneration after PHx compared with 2- to 3-month-old mice (67). Although the expression of p21 persisted longer in hepatocytes of 6- to 8-month-old mice compared with young mice, these cells do not exhibit the full senescence phenotype. Whereas the senolytic drug ABT737 did not induce apoptosis in these hepatocytes, it nevertheless reduced the expression of p21 and some components of the SASP through unknown mechanisms, resulting in improved liver regeneration after PHx (67). These results suggest that age-related damage in hepatocytes can lead to multiple cellular defects, some of which overlap with the senescence phenotype, and expression of the cell cycle inhibitor p21 in hepatocytes may impede liver regeneration.

To minimize the effects of age-related defects, we have focused on young mice in this study. Surprisingly, we found that senescent HSCs rapidly emerged as a programmed response to PHx in young mice with a time course concomitant with that of hepatocyte proliferation. Here we show that the elimination of senescent cells by different approaches, including ABT263 treatment and the p16-3MR model, resulted in impaired liver regeneration and delayed hepatocyte proliferation. Each of these models resulted in diminished activation of STAT3, SFKs, YAP, and ERK1/2 in the liver (Figure 3, B and C). Senescent HSCs secreted IL-6 and CXCR2 ligands as part of the SASP to promote hepatocyte proliferation and liver regeneration (Figure 6E). Elimination of senescent cells greatly delayed liver weight restoration after PHx, and these defects could be fully mitigated by the administration of IL-6 and CXCL2, one of the CXCR2 ligands expressed in senescent HSCs (Figure 5). Of note, the vast majority of senescent cells that emerged in the liver in young mice after PHx were derived from HSCs (>87%), and only about 7% of senescent cells were hepatocytes (Figure 1E and Supplemental Figure 1E). As hepatocytes far outnumber HSCs, only a small fraction of hepatocytes may experience senescence-induced growth arrest. The prevalence of senescence in parenchymal cells will undoubtedly compromise regeneration by inhibiting their proliferation. Indeed, senescence induced by cell type–specific deletion of *Mdm2* in hepatocytes or cholangiocytes resulted in impaired regeneration (19, 20). Senescence is known to alter the transcriptome and secretome of HSCs (34, 35), including the expression of IL-6 and CXCR2 ligands (Figure 1, D and H, and Supplemental Figure 2C). However, the elimination of senescent cells resulted in no difference in the number of macrophages and neutrophils in the liver (Supplemental Figure 1, C and D), suggesting that the overall effects of the senescent cells on inflammatory cells in PHx are minimal.

The importance of IL-6 in liver regeneration has long been appreciated, as deletion of *Il-6* or blockade of IL-6 signaling delays liver regeneration and decreases survival after PHx (38, 68). A large amount of IL-6 is found in the circulation within 2 hours after PHx before the initiation of cell proliferation, priming hepatocytes to respond to mitogens such as HGF and EGF (2, 69). Whereas Kupffer cells are the major source of IL-6 in this early expression (70), our findings show that IL-6 was also expressed 2 days after PHx during the proliferative phase of liver regeneration and this expression was largely driven by senescent cells (Figure 3A). Likewise, the expression of the CXCR2 ligands, including CXCL2, was also senescent cell dependent. IL-6 activated multiple downstream pathways to enhance hepatocyte proliferation, including STAT3 and YAP, and synergized with CXCL2 to induce ERK1/2 activation (Figure 2H and Figure 4D). Interestingly, the crosstalk between IL-6 and CXCL2 resulted in repression of AKT activation. The robust activation of YAP, STAT3, and ERK1/2 by IL-6 and CXCL2 after PHx may explain how these factors can fully restore liver regeneration in mice with senescent cell elimination (Figure 4D).

Mice with liver-specific deletion of *Yap* or *Yap* and *Taz* suffer significantly blunted liver weight restoration after PHx (55, 56), similar to IL-6–knockout mice (39). By contrast, the effect of liver-specific *Stat3* deletion was relatively modest (71, 72), suggesting that *Yap* may play a more important role than *Stat3* in liver regeneration. Although the canonical regulation of YAP is through the Hippo pathway, our results show that YAP activation at the peak of hepatocyte proliferation was driven by senescent HSCs through IL-6–induced SFK activation (Figures 2 and 3 and Supplemental Figure 6, B–E). The regulation of YAP by cellular senescence is previously unknown, we believe, and may highlight a unique mechanism of YAP activation in the context of injury and regeneration.

The specific signals that trigger injury-induced senescence may vary depending on the etiology. In the context of PHx, the matricellular protein CCN1 is quickly elevated and leads to HSC senescence (Figure 6). We have previously shown that CCN1 induces HSC senescence in CCl_4_-induced inflammatory injury in the liver and limits fibrosis (22). Mechanistically, CCN1 induces senescence by binding to integrin α_6_β_1_ to trigger a DNA damage response through p53- and p16^INK4a^/pRb pathways (16). Although CCN1 is normally expressed at a low level in most cell types, it is released from α-granules of platelets upon vascular injury and platelet activation (62). The number of senescent cells was reduced by about 75% in *Ccn1^DM/DM^* mice after PHx compared with controls (Figure 6C), indicating that CCN1 is the principal inducer of HSC senescence. By comparison, ABT263 treatment was more effective in eliminating SA-β-Gal^+^ senescent cells (>90%) and accordingly resulted in more severe impairment in liver weight restoration. The importance of CCN1/α_6_β_1_-induced senescence in regeneration is consistent with the observation that knockdown or knockout of integrin β_1_ impairs liver regeneration (73). However, since integrin β_1_ can interact with multiple α subunits to form 12 distinct integrin heterodimers (74), its functions through other mechanisms in PHx cannot be excluded. Interestingly, *Ccn1^DM/DM^* mice showed impaired activation of YAP, STAT3, and SFKs but did not suffer a loss of ERK1/2 activation after PHx (Figure 6B and Supplemental Figure 8D), and correspondingly, enjoyed a faster remnant liver recovery than other models of senescent cell elimination. The compensatory mechanism that allowed ERK1/2 activation despite the loss of senescent cells in *Ccn1^DM/DM^* mice is currently unknown. However, ERK1/2 can be activated by multiple signaling pathways. Since the CCN1-DM mutant is unable to bind integrin α_6_β_1_, we speculate that it may be more likely to interact with other receptors, including α_v_β_3_ and α_M_β_2_, in other cell types and stimulate alternative signaling that directly or indirectly results in ERK1/2 activation in hepatocytes (75).

*Ccn1* is also a YAP target gene often used as a readout for YAP-dependent transcription because of its strong response to YAP activity (76). Thus, *Ccn1* and *Yap* are in a regulatory loop in which CCN1 activates YAP through senescence-mediated IL-6 expression, and YAP transcriptionally activates *Ccn1*. A deficit in senescent cells induced by ABT263 or in *Ccn1^DM/DM^* mice results in decreased YAP activation, which in turn leads to curtailed *Ccn1* expression (Supplemental Figure 6A and Supplemental Figure 8C). Intriguingly, CCN1 has also been shown to exert negative feedback to YAP in endothelial cells, where *Ccn1* overexpression inactivates YAP by altering cell-matrix signaling (77). Thus, CCN1 may regulate YAP activation positively or negatively in a context-dependent manner.

Although the liver regenerates to its original size after PHx, the biliary tree is not regenerated. Instead, the bile ducts are enlarged to accommodate the bile produced in the regenerated liver. CCN1 is known to play a prominent role in biliary regeneration in response to ductular reaction after cholestatic damage (78). In response to cholestasis, CCN1 induces the activation of NF-κB through interaction with integrins α_v_β_3_/α_v_β_5_ to induce the expression of *Jag1*, which in turn activates NOTCH1 to induce cholangiocyte proliferation (78). Thus, CCN1 may trigger cholangiocyte proliferation to promote bile duct expansion after PHx. If so, CCN1 may act through 2 distinct mechanisms in liver regeneration in response to PHx: i) induction of HSC senescence through integrin α_6_β_1_ to promote hepatocyte proliferation and ii) activation of JAG1/NOTCH1 signaling through integrins α_v_β_3_/α_v_β_5_ to promote cholangiocyte proliferation. A detailed assessment of the role of CCN1 in bile duct expansion after PHx will require further investigation.

The unique regenerative capacity of the liver allows surgical resection or liver transplantation as treatment for hepatocellular carcinoma and advanced cirrhosis. Our study suggests that senescent cells may be important for optimal liver regeneration in some patients. As the clearance of senescent cells by senolytic drugs is being contemplated for treating a variety of pathologies (79–81), the potential effects of eliminating senescent cells in the context of tissue regeneration and repair warrant careful consideration.

## Methods

### Animals and surgery.

*Ccn1^DM/DM^* mice were developed in our laboratory and backcrossed to C57BL/6 (The Jackson Laboratory) more than 20 times (82). p16-3MR mice were provided by Unity Biotechnology (17). PHx was carried out under general anesthesia as previously described (83). Sham-operated animals underwent abdominal surgery without liver resection. Livers were collected at indicated times after surgery, and the remnant liver weight recovery rate was calculated using the following equation:
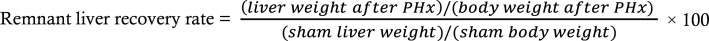


### Isolation of hepatic cells.

Hepatic cells were isolated as described with minor modifications (84, 85). Briefly, livers from 2- to 3-month-old mice were perfused with a digestion solution of L-15 medium (Gibco) with collagenase, elastase, and DNase mixture (Worthington Biochemical). The liver tissues were gently shaken to release the hepatic cells, and the cell suspension was sequentially filtered through 100 μm, 70 μm, and 40 μm cell strainers (all from Thermo Fisher Scientific) to remove cell clusters and centrifuged at 30*g* for 3 minutes at 4°C. After centrifugation, the hepatocytes formed a pellet and nonparenchymal cells remained in suspension. The pellet was resuspended in cold L-15 medium and washed 3 times by centrifugation at 30*g* for 3 minutes at 4°C. The hepatocyte pellets were resuspended and plated at 3 × 10^5^ cells/mL in culture dishes precoated with 0.1% mouse tail collagen (Electron Microscopy Science) followed by incubation in fresh serum-free William E medium (Thermo Fisher Scientific).

To isolate HSCs, the supernatant fractions containing nonparenchymal cells mentioned above were collected and centrifuged for 650*g* for 3 minutes at 4°C to pellet the cells. The cell pellets were resuspended in 10 mL cold HBSS containing 0.5% BSA and were transferred on the top of 30 mL 11.5% Nycodenz (11.5% w/v; Accurate Chemical) in a 50 mL tube and centrifuged at 1500*g* for 22 minutes at 4°C. After centrifugation, HSCs forming a white band at the midphase of the Nycodenz were collected, resuspended in HBSS containing 0.5% BSA, and washed before resuspension in prewarmed DMEM containing 10% FBS. Cells thus isolated were approximately 90% positive for oil red O staining for lipid droplets and desmin staining, confirming their identity as HSCs (Supplemental Figure 2A).

### Oil red O staining.

Primary HSCs were fixed with 10% formaldehyde for 10 minutes at room temperature. After washings with PBS, cells were rinsed in 60% isopropanol and incubated with oil red O working solution (MilliporeSigma) for 20 minutes at room temperature. After incubation, cells were washed 3 times with 60% isopropanol and counterstained with hematoxylin.

### Treatment with inhibitors and recombinant proteins.

Mice were injected i.p. with ABT263 (100 mg/kg; Selleckchem), GCV (30 mg/kg; Selleckchem), SCH772984 (25 mg/kg; Selleckchem), PP2 (5 mg/kg; MilliporeSigma), IL-6 (50 μg/kg; PeproTech), CXCL2 (15 μg/kg; R&D Systems), or vehicles at the indicated times as previously described (45, 67). Primary hepatocytes were attached to collagen-coated dishes in serum-free medium and treated with recombinant IL-6 (10 ng/mL) or CXCL2 (10 ng/mL) for 24 hours. Where indicated, primary cells were pretreated with the ERK1/2 inhibitor SCH772984 (1 μM), STAT3 inhibitor WP1066 (5 μM; Avantor), YAP inhibitor Super-TDU (50 nM; Selleckchem), or AKT inhibitor MK2206 (1 μM; Selleckchem) for 1.5 hours prior to experiments. The inhibition of ERK1/2, STAT3, and AKT was confirmed by Western blots. The effect of Super-TDU was confirmed by the reduced expression of YAP target genes in IL-6–treated hepatocytes (Supplemental Figure 5, C–E).

### RNA interference.

Senescent HSCs were transfected with 50 nM siRNA targeting *IL-6* or a nontargeting control siRNA, and primary hepatocytes were transfected with 50 nM siRNA targeting *Cxcr2* or a nontargeting control siRNA (Horizon: siGENOME SMARTpool system) according to manufacturer’s protocol. siRNA sequences are shown in Supplemental Table 1. Gene expression was analyzed by qRT-PCR 48 hours after transfection.

### Coculture of senescent HSCs and hepatocytes.

To generate senescent HSCs, primary HSCs were treated with MMC (10 μg/mL; MilliporeSigma) for 3 hours at 37°C. After incubation, cells were washed 3 times with prewarmed PBS and cultured with DMEM + 10% FBS for 3 days. Cellular senescence was confirmed by proliferation arrest, morphological changes, SA-β-Gal staining, and p16 and SASP expression (Supplemental Figure 4, A–C). Before coculturing, primary hepatocytes were seeded in 12-well plates with collagen-coated coverslips (Electron Microscopy Sciences CAT 72295). The HSCs or senescent HSCs were seeded in the Transwell coculture baskets (0.4 μm pores; Costar). After cell attachment and PBS wash steps, the Transwell inserts with naive or senescent HSCs were placed onto the 12-well plates containing hepatocytes and incubated in serum-free William E medium at 37°C for 24 hours. Where indicated, monoclonal anti–IL-6 antibody (catalog 16-7061-81; 5 μg/mL; Invitrogen) or SB225002 (1 μM; Selleckchem) was added to the 12-well plates before the inserts were placed. The hepatocytes were fixed by formalin and made membrane permeable with PBS + 0.1% Triton X-100, then stained with anti-Ki67 (Abcam).

### SA-β-Gal and MPO assays.

SA-β-Gal assay was performed as described (22). The primary cells or frozen tissue sections (6 μm) were fixed with 0.5% glutaraldehyde in PBS for 10 minutes. After washings with PBS to remove the glutaraldehyde, the reaction solution (5 mM potassium ferricyanide, 5 mM potassium ferrocyanide, 1 mg/mL of X-gal, 1 mM magnesium chloride in pH 5.5 PBS) was added and incubated overnight at 37°C. The tissue sections were counterstained by eosin. MPO assay was performed using an assay kit (BioVison) according to the manufacturer’s protocol.

### Immunoblotting, ELISA, and serum biochemistry.

Western blot analyses were performed using standard procedures with the ECL Plus system (Thermo Fisher Scientific). Antibodies targeting the following proteins were used: p-YAP (Y357) (Abcam, ab62751), YAP (Cell Signaling Technology, 4912), p-STAT3 (Y705) (Cell Signaling Technology, 9145), STAT3 (Cell Signaling Technology, 12640), p-SFKs (Y416) (Cell Signaling Technology, 2101), SRC (Thermo Fisher Scientific, AHO1152), Yes (Cell Signaling Technology, 65890), AKT1 (Cell Signaling Technology, 2938), p-AKT (S473) (Cell Signaling Technology, 4060), p38 (Cell Signaling Technology, 8690), p-p38 (T180/Y182) (Cell Signaling Technology, 4511), ERK1/2 (Cell Signaling Technology, 4695), p-ERK1/2 (T202/Y204) (Cell Signaling Technology, 4370), CCN1 (R&D Systems, AF4055), and β-actin (Abcam, ab8226) were detected by the antibodies. The intrahepatic IL-6 and CXCL2 levels were analyzed in the whole liver lysates by using an IL-6 ELISA kit (eBioscience) and CXCL2 ELISA kit (R&D Systems). The serum levels of ALT and AST were measured by the Biological Resources facility at the University of Illinois at Chicago using a Beckman Coulter AU480 analyzer.

### Immunohistochemistry and immunofluorescence.

For immunohistochemistry, tissues were formalin fixed and embedded in paraffin after dehydration. After preparation of 5 μm sections, the epitopes were unmasked by pH 6.0 sodium citrate, then incubated with the primary antibodies against Ki67 (Abcam, ab16667), Ly6G (BD Biosciences, 551459), SOX9 (MilliporeSigma, ab5535), YAP (Cell Signaling Technology, 4912), p-ERK1/2 (Cell Signaling Technology, 4370), or p-STAT3 (Cell Signaling Technology, 9145). For immunofluorescence, tissues were formalin fixed and sucrose processed and embedded in Optimal Cutting Temperature Embedding Medium (Thermo Fisher Scientific). After preparing 6 μm frozen sections, the epitopes were unmasked by 0.1% sodium borohydride and allowed to become membrane permeable by PBS with 0.1% Triton X-100, then incubated with primary antibodies against p16 (Santa Cruz Biotechnology, sc-1661), albumin (Abcam, ab19194), desmin (Abcam, ab32362), F4/80 (AbD Serotec, MCA497RT), RFP (Abcam, ab185921), or cleaved caspase-3 (Cell Signaling Technology, 9661).

### RNA isolation and qRT-PCR.

The total RNA was isolated from liver tissues and cultured cells using RNeasy Mini Kit (Qiagen) following the manufacturer’s protocol. The cDNA was prepared by reverse transcription of purified RNA using Superscript Reverse Transcriptase III (Invitrogen). qRT-PCR was performed by mixing the cDNA and the primers of the target genes in iQ SYBR Green Supermix (Bio-Rad). PCR primer sequences are shown in Supplemental Table 2. The reactions were carried out in iCycler Thermal Cycler (Bio-Rad), and the specificity was confirmed by melting curve analysis.

### Statistics.

All experiments were carried out with at least 3 biological replicates. Student’s 2-tailed *t* tests were used to compare the difference between the 2 groups. For multiple groups’ comparisons, 2-way ANOVA and Bonferroni’s post hoc test were used. *P* < 0.05 was considered significant. Statistical analysis was performed with GraphPad Prism 9 software.

### Study approval.

All animal protocols were approved by the Institutional Animal Care and Use Committee of the University of Illinois at Chicago.

## Author contributions

NC, KHK, and LFL designed the experiments and analyzed the data; NC and KHK performed the experiments; and NC and LFL wrote the manuscript.

## Supplementary Material

Supplemental data

## Version 1 06/16/2022

In-Press Preview

## Version 2 07/22/2022

Electronic publication
