# Supplementary material for: Senescent hepatic stellate cells promote liver regeneration through IL-6 and ligands of CXCR2
Source: JCI Insight. 2022 Jun 16;7(14):e158207. doi: 10.1172/jci.insight.158207 (PMC9431681; doi:10.1172/jci.insight.158207)
Supplement: Supplemental data [file jciinsight-7-158207-s008.pdf]

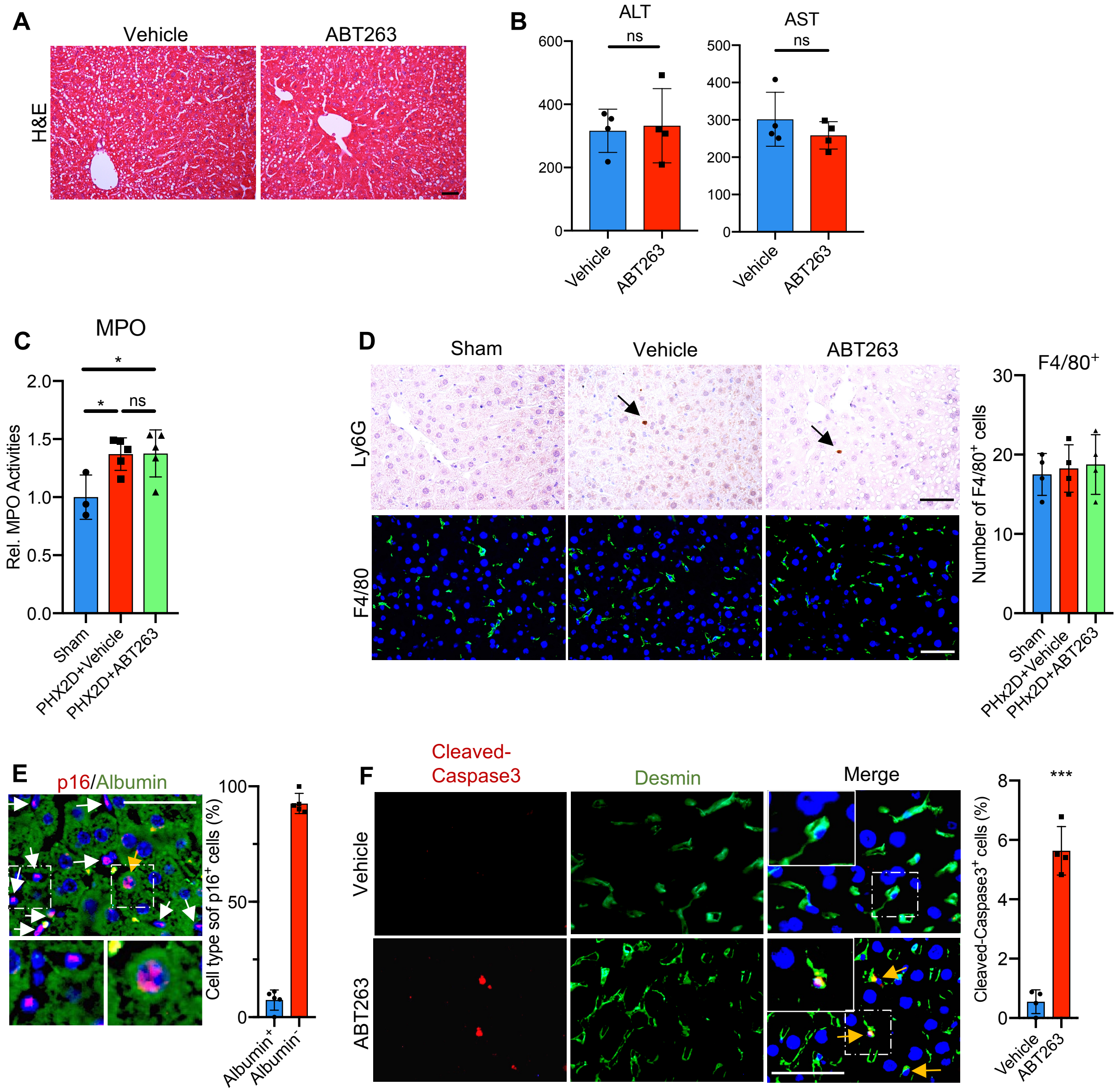

Supplemental Figure 1. Vehicle- and ABT263-treated mice sustained similar liver damage after PHx. (A) H&E staining of liver tissue sections from vehicle- or ABT263-treated WT mice 2 days post-PHx. (B) Serum levels of ALT and AST were analyzed (*n=4*). (C) MPO activity in mice was measured 2 days post PHx (*n=3* for sham control, *n=5* for vehicle- and ABT263-treated mice). (D) Liver sections of sham-operated mice or mice subjected to PHx with or without ABT263 exposure were stained with anti-Ly6G or anti-F4/80 antibodies 2 days after surgery. Arrows point to Ly6G^{+} cells. The number of F4/80^{+} cells per microscopic field was quantified by cell counting (n=4). (E) Liver sections from WT mice 2 days after PHx were immunostained for p16 (red) and Albumin (green) and counterstained with DAPI (blue). The percentages of p16^{+} cells that were Albumin^{+} (yellow arrow and lower right panel) or Albumin^{-} (white arrows and lower left panel) were quantified by cell counting (*n=5*). (F) Liver sections of vehicle- and ABT263-treated WT mice 2 days post-PHx were stained with anti-cleaved caspase3 (red) and anti-Desmin (green) antibodies and counterstained with DAPI (blue). Yellow arrows point to cleaved-Caspases3^{+} cells. The percentage of Caspase 3^{+} cells were quantified by cell counting (*n=4*). Scale bar: 50 µm. Data expressed as mean +/- SD. **P*<0.033, ***P*<0.002, ****P*<0.001, Student's *t*-test.

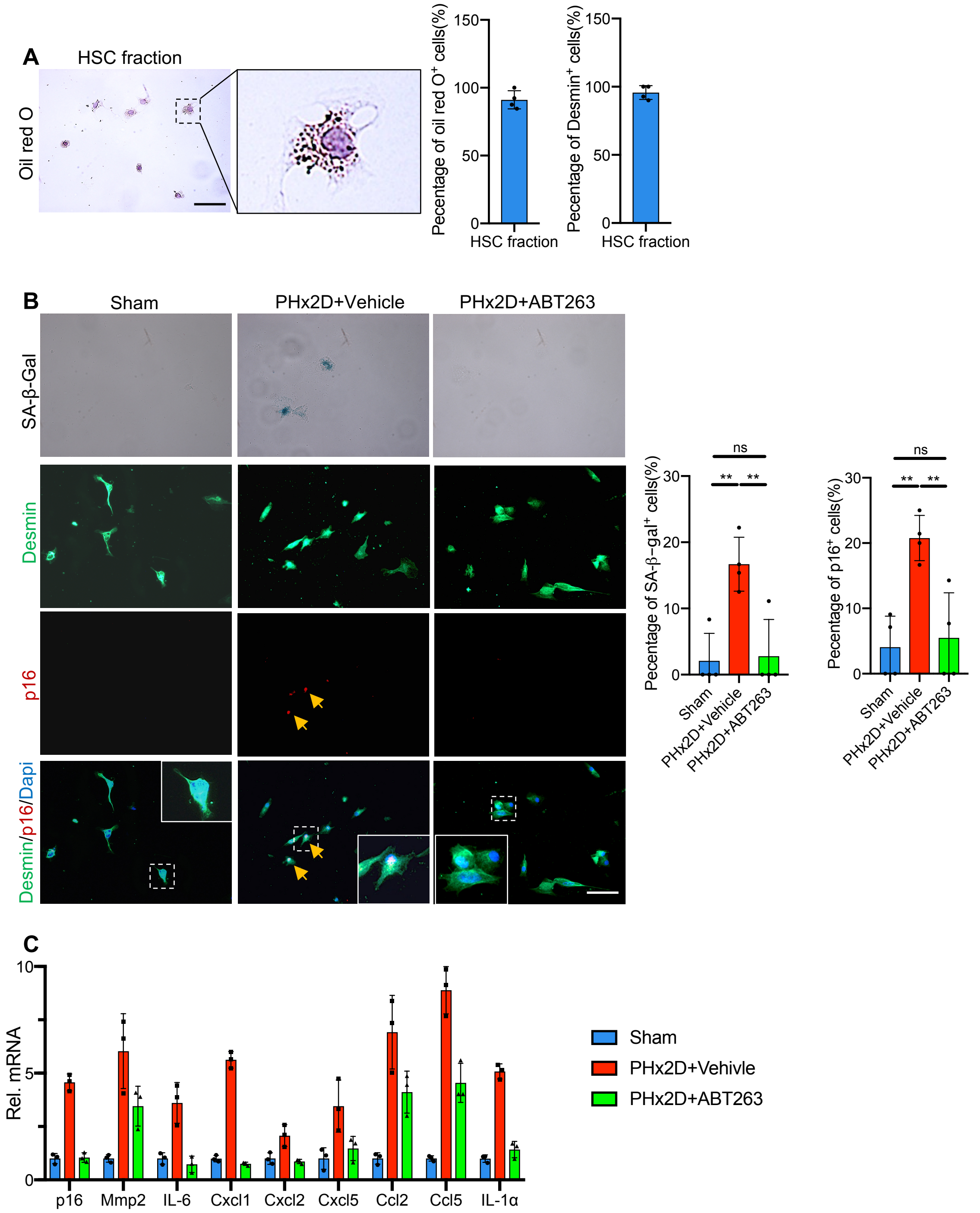

Supplemental Figure 2. PHx induces HSC senescence. (A) After liver perfusion and gradient centrifugation, primary cells from the HSC fraction were stained with oil red O and counterstained with Hematoxylin. The percentage of oil red O⁺ cells and Desmin⁺ cells were quantified by cell counting (*n=4*). (B) Primary HSCs isolated from indicated livers 2 days after PHx were stained for SA-β-Gal activity (blue) or were double immunostained for p16 (red) and Desmin (green) and counterstained with DAPI (blue). Yellow arrows point to p16⁺ cells. The number of SA-β-Gal⁺ cells and p16⁺ were quantified by cell counting (*n=4).* (C) Expression of *p16* and genes of the SASP in the primary HSCs isolated from the indicated livers was quantified by qPCR (*n*=3). Scale bar: 50 μm. Data expressed as mean +/- SD. **P*<0.033, ***P*<0.002, ****P*<0.001, Student's *t*-test.

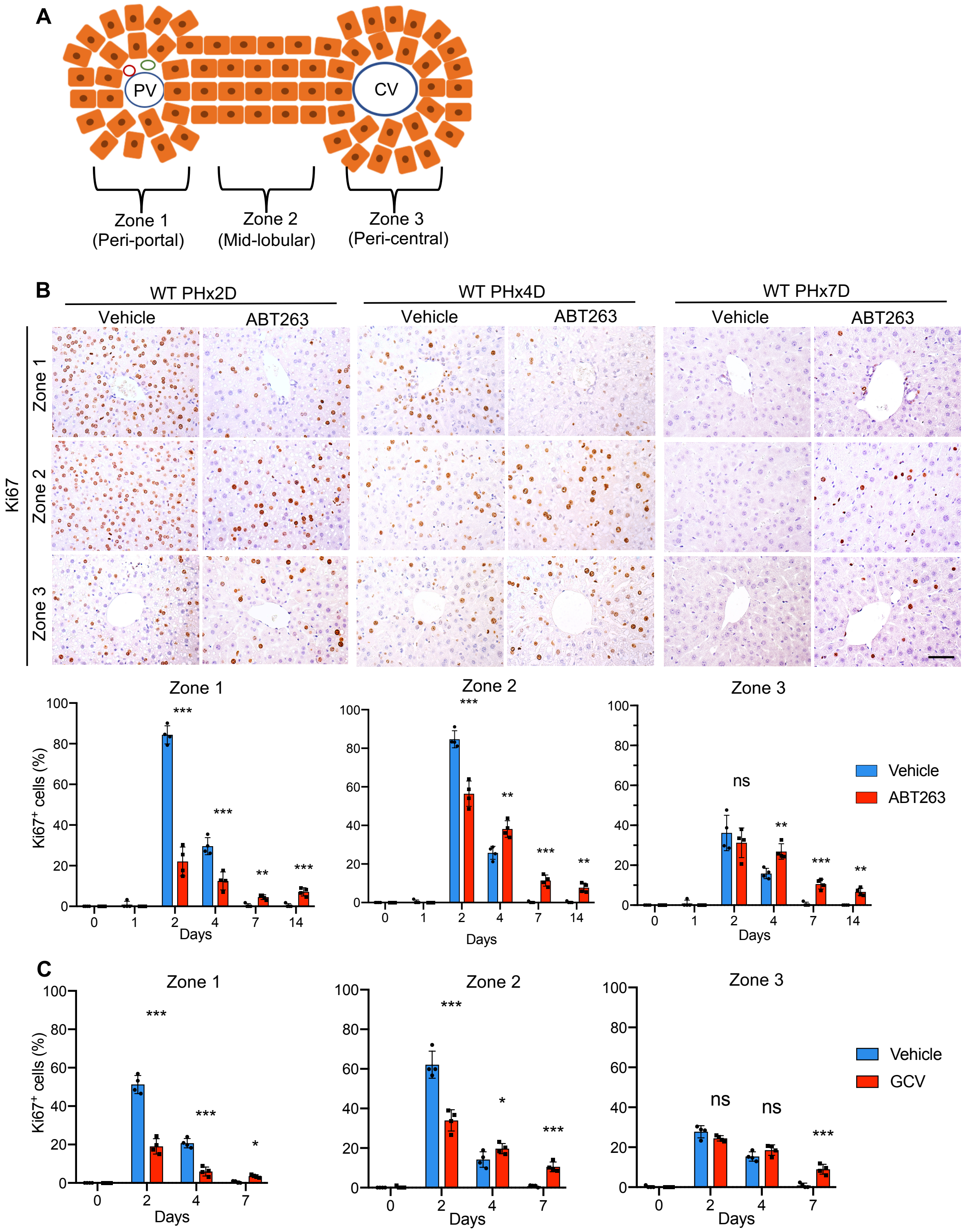
A
PV
CV
Zone 1
(Peri-portal)
Zone 2
(Mid-lobular)
Zone 3
(Peri-central)
B
WT PHx2D
WT PHx4D
WT PHx7D
Vehicle
ABT263
Ki67
Zone 1
Zone 2
Zone 3
Ki67+ cells (%)
Days
Vehicle
ABT263
ns
C
Zone 1
Zone 2
Zone 3
Ki67+ cells (%)
Days
Vehicle
GCV
ns
ns

Supplemental Figure 3. Reduced hepatocyte proliferation in livers of mice with senescent cell elimination. (A) Schematic diagram of the hepatic zones: zone 1 (peri-portal region), zone 2 (mid-lobular region), and zone 3 (peri-central region) are indicated. (B) Livers from WT mice treated with vehicle or ABT263 were harvested at 0, 1, 2, 4, 7, and 14 days after-PHx and were sectioned and stained with anti-Ki67 antibodies. Staining of liver sections from days 2 (PHx2D), 4 (PHx4D), and 7 (PHx7D) in zones 1, 2, and 3 are shown. The number of $Ki67^+$ hepatocytes was quantified by cell counting (*n=4*). (C) Livers from p16-3MR mice treated with vehicle or GCV were harvested at 0, 2, 4, and 7 days post-PHx and were sectioned and stained with anti-Ki67 antibodies. The number of $Ki67^+$ hepatocytes was quantified by cell counting (*n=4*). Scale bar: 50 μm. Data expressed as mean +/- SD. *$P<0.033$, **$P<0.002$, ***$P<0.001$, Student's *t*-test.

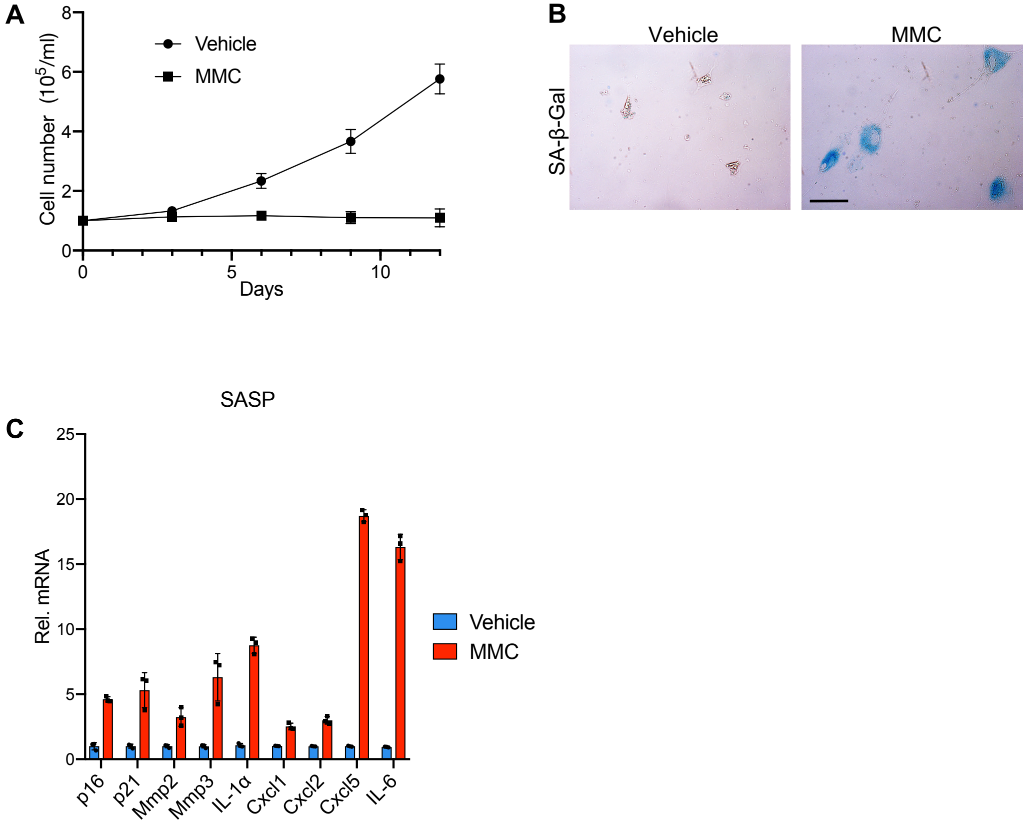

Supplemental Figure 4. Characterization of MMC-induced senescent HSCs.
(A) Proliferation of vehicle- and MMC- treated primary HSCs was assessed by cell counting (*n*=3). (B) Primary HSCs treated with vehicle or MMC to induce senescence were stained for SA-β-Gal activity. (C) Expression of senescence-related genes and genes of the SASP in HSCs was measured by qPCR analysis (*n*=3). Scale bar: 50 μm. Data expressed as mean +/- SD. **P*<0.033, ***P*<0.002, ****P*<0.001, Student's *t*-test.

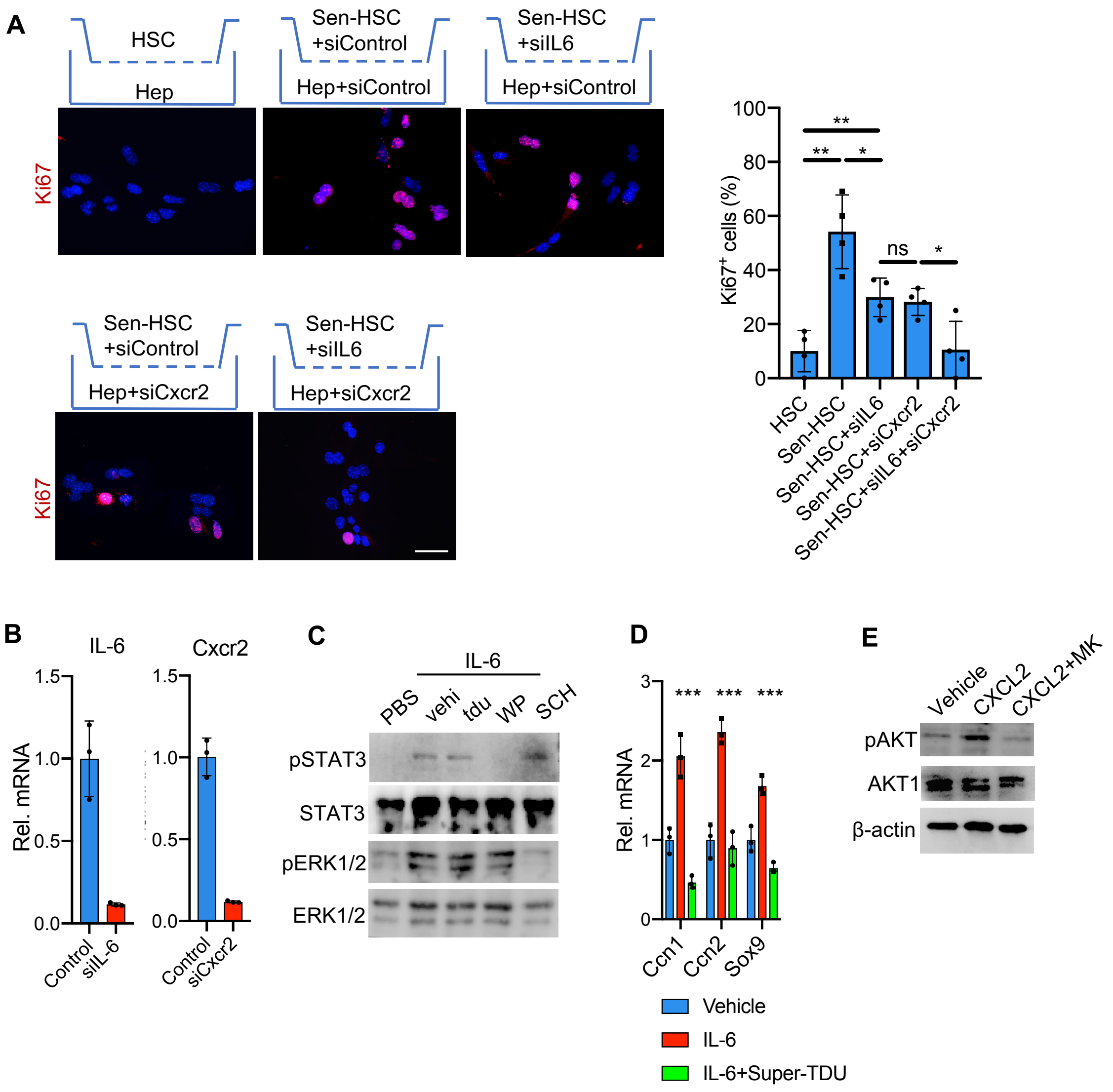

Supplemental Figure 5. IL-6 and CXCR2 ligands of senescent HSCs induce hepatocyte proliferation.

(A) Primary hepatocytes isolated from 2-month-old WT male livers were treated with si*Control* or si*CXCR2* then co-cultured with HSCs, senescent HSCs (Sen-HSC), and Sen-HSC which were treated with si*Control* or si*IL-6* as indicated. After 24 hrs, primary hepatocytes were stained with anti-Ki67(red). The number of Ki67^{+} cells was quantified by cell counting *(n=4)*. (B) The expression of *IL-6* in Sen-HSCs and *Cxcr2* in hepatocytes was quantified by qPCR *(n=3).* (C) Hepatocytes were treated with vehicle, IL-6, Super-TDU (TDU; 50 nM), WP1066 (WP; 5 μM) and SCH772984 (SCH; 1 μM) as indicated for 24 hrs were probed with indicated antibodies by immunoblotting. (D) The expression of YAP target genes in indicated hepatocytes was quantified by qPCR (*n=3*). (E) Hepatocytes were treated with vehicle, CXCL2, MK2206 (MK; 1 μM) as indicated for 24 hrs and were probed with indicated antibodies by immunoblotting. Scale bar: 50 μm. Data expressed as mean +/- SD. **P*<0.033, ***P*<0.002, ****P*<0.001, Student's *t*-test.

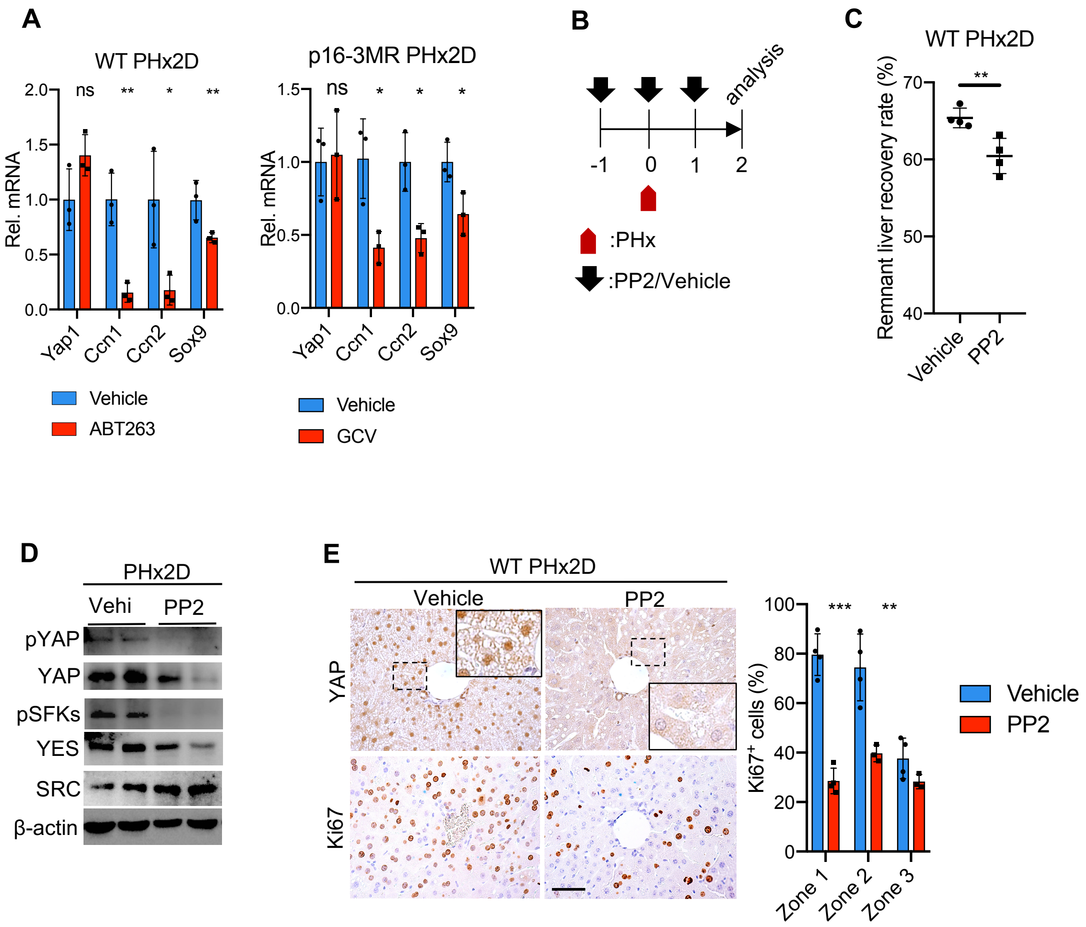

Supplemental Figure 6. Inhibition of SFKs impairs YAP activation and liver regeneration. (A) In livers of WT or p16-3MR mice 2 days post-PHx with and without injection of ABT263 or GCV, respectively, the expression of *Yap1* and YAP target genes (*Ccn1*, *Ccn2*, *Sox9*) was quantified by qPCR (*n=3*). (B) Experimental scheme of WT male mice subjected to PHx with or without injections of PP2 (5 mg/Kg). (C) The remnant liver recovery rates in vehicle- or PP2-treated WT mice were assessed two days post PHx (*n=4*). (D) Whole liver lysates from indicated mice 2 days post-PHx were immunoblotted and probed with antibodies against pYAP (Y357), YAP, pSFKs (Y416), YES, SRC and β-actin. (E) Liver tissue sections of the indicated livers were stained with anti-Ki67 and anti-YAP antibodies; zone 1 is shown. The number of $Ki67^{+}$ cells in different zones was analyzed by cell counting (n=3). Scale bar: 50 μm. Data expressed as mean +/- SD. **P*<0.033, ***P*<0.002, ****P*<0.001, Student's *t*-test.

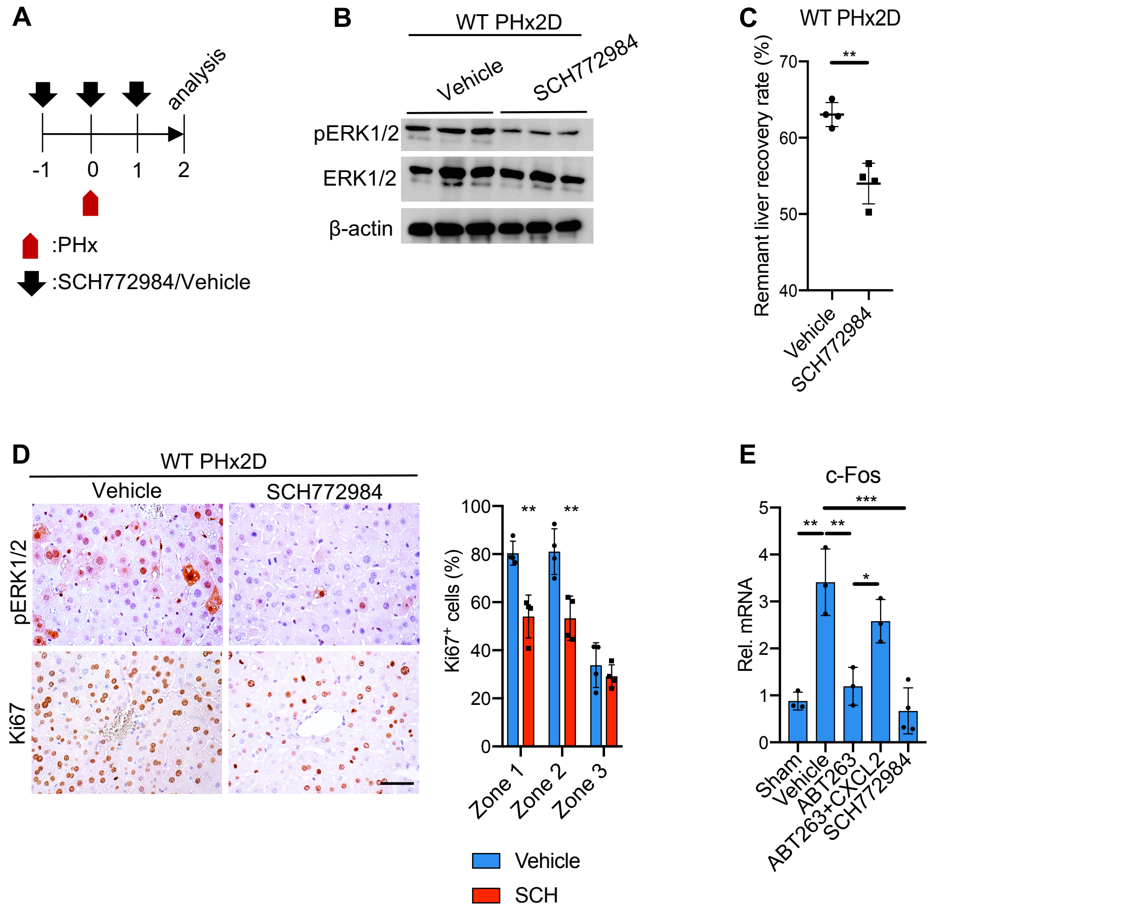

Supplemental Figure 7. SCH772984 inhibits ERK1/2 activation and impairs liver regeneration.

(A) Experimental scheme of WT male mice subjected to PHx with or without injections of SCH772984 (25 mg/Kg). (B) Whole liver lysates from vehicle-treated and SCH772984-treated WT mice two days post-PHx were immunoblotted and probed with anti-pERK1/2, anti-ERK1/2, and anti-β-actin antibodies. (C) The remnant liver recovery rates of WT mice with or without SCH772984 treatment were measured 2 days post-PHx (*n=4*). (D) Liver sections of the above were stained for anti-pERK1/2, and anti-Ki67 antibodies. The Ki67$^+$ cells in all three zones were quantified by cell counting. (E) The mRNA expression levels of *c-fos* in the indicated livers two days after PHx were detected by qPCR (*n=4* for SCH772984, *n=3* for the others.) Scale bar: 50 µm. Data expressed as mean +/- SD. **P*<0.033, ***P*<0.002, ****P*<0.001, Student's *t*-test.

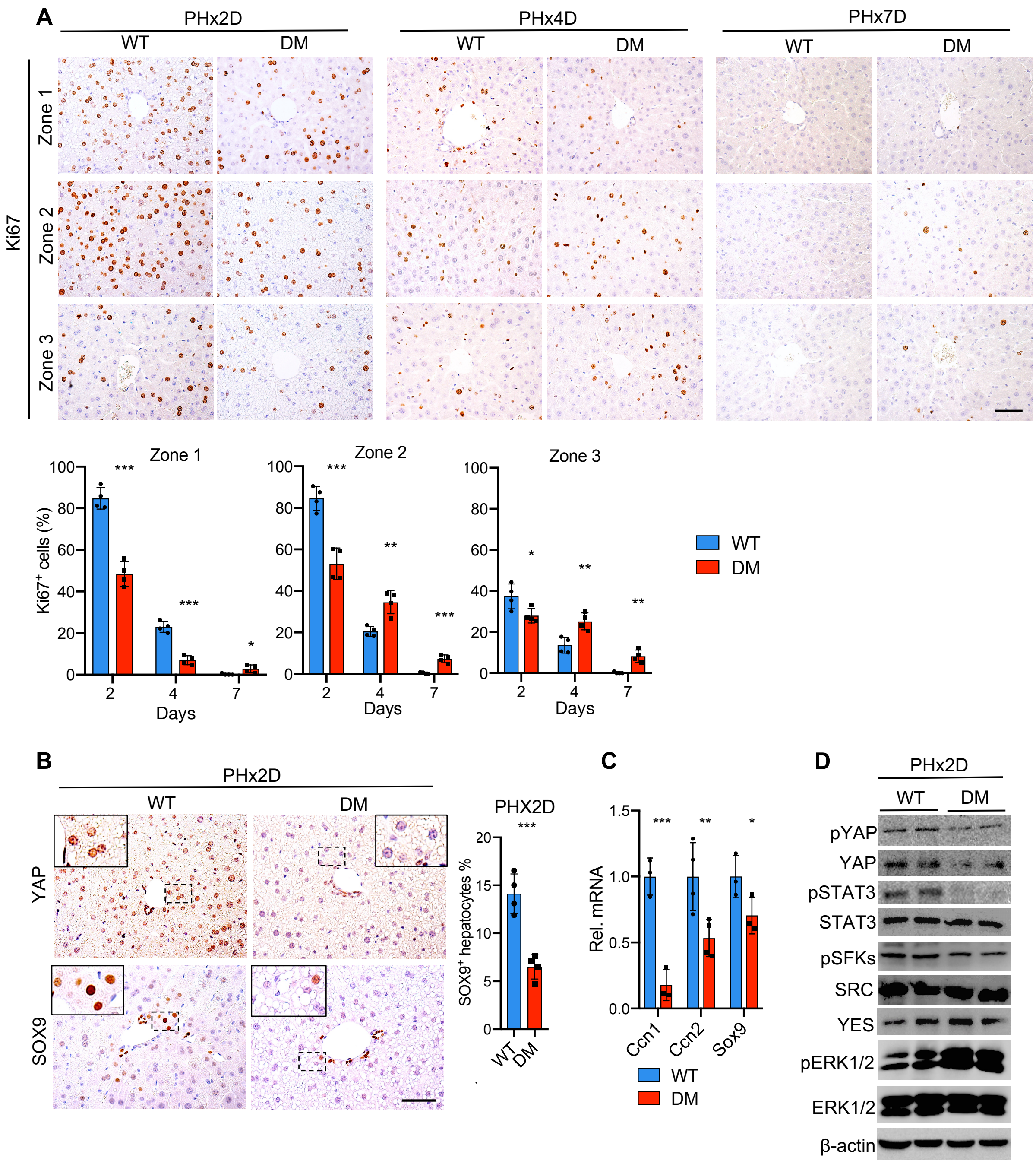

Supplemental Figure 8. ***Ccn1*** $^{DM/DM}$ **mice exhibit impaired regenerative responses.**
(A) Liver sections of WT and *Ccn1* $^{DM/DM}$ (DM) mice collected at 2, 4, and 7 days post-PHx were stained with anti-Ki67 antibodies. Ki67⁺ cells were quantified by cell counting (*n*=4). (B) Liver sections were also stained with anti-YAP and anti-SOX9 antibodies; zone 1 is shown. The number of SOX9⁺ hepatocytes was quantified by cell counting (*n=4*). (C) The expression levels of *Ccn1*, *Ccn2*, and *Sox9* in WT and DM livers two days post-PHx were assessed by qRT-PCR (*n*=3). (D) Whole liver lysates of these livers were probed with antibodies against pYAP (Y357), YAP, pSTAT3 (Y705), STAT3, pSFKs (Y416), SRC, YES, pERK1/2 (T202/Y204), ERK1/2 and β-actin. Scale bar: 50 μm. Data expressed as mean +/- SD. **P*<0.033, ***P*<0.002, ****P*<0.001, Student's *t*-test.

Gene	Target sequence (5’ to 3’)
Cxcr2-1	CAGUGGAGAUCUUGAUAUU
Cxcr2-2	UAAAGUAAAUGGAUGGACU
Cxcr2-3	CGAAAUCCUGUUAAGGUAA
Cxcr2-4	AAUUCAAGGUGGAUAAGUU
IL-6-1	CCAAUGCUCUCCUAACAGA
IL-6-2	GGAUACCACUCCCAACAGA
IL-6-3	CUACCAAACUGGAUAUAAU
IL-6-4	GAUCUACUCGGCAAACCUA

Supplemental Table 1. siRNA sequences used in this study

Gene name	Forward primer (5' to 3')	Reverse primer (5' to 3')
p16	AATCTCCGCGAGGAAAGC	GTCTGCAGCGGACTCCAT
p21	CAGATCCACAGCGATATCCAG	AGAGACAACGGCACACTTTG
Mmp2	TAACCTGGATGCCGTCGT	TTCAGGTAATAAGCACCCTTGAA
Mmp3	CAAAACATATTTCTTTGTAGAGGACAA	TTCAGCTATTTGCTTGGGAAA
Mmp12	TGAATTTGCATTTCCTGTACATAGT	TGCTGTAAGTCCATGGGTGA
Cxcl1	AACCGAAGTCATAGCCACAC	CAGACGGTGCCATCAGAG
Cxcl2	GAAGTCATAGCCACTCTCAAGG	CTTCCGTTGAGGGACAGC
Cxcl5	GTTCCATCTCGCCATTCATG	TTAAGCAAACACAACGCAGC
Pai-1	AGGATCGAGGTAAACGAGAGC	TTGGTTGAGGGAATCATTCAT
Ccl2	CATCCACGTGTTGGCTCA	GATCATCTTGCTGGTGAATGAGT
Ccl5	TGCAGAGGACTCTGAGACAGC	GAGTGGTGTCCGAGCCATA
IL1-α	TCCATAACCCATGATCTGGAA	TTGGTTGAGGGAATCATTCAT
IL-6	ACCACTCCCAACAGACC	TCCAGAAGACCAGAGGAA
Ccn1	AAAGGCAGCTCACTGAAG	GCCGGTATTTCTTGACAC
Ccn2	TAGGCCCTCAGCCTCACT	CTTGACAGGCTTGGGGAT
Sox9	GCTTTTCGAATACTGCAAACTCC	CGCAAGTGTGTGTGTCTAGACT
Yap1	ACCAATAGTTCCGATCCCTTTC	TGTCTCCTGTATCCATTTCATCC
c-fos	CAGCCTTTCCTACTACCATTCC	GGATAAAGTTGGCACTAGAGACG
Cxcr2	GCTCACAAACAGCGTCGTAGAA	GAGTGGCATGGGACAGCATC
Cyclophilin E	TTCACAAACCACAATGGCACAGGG	TGCCGTCCAGCCAATCTGTCTTAT

Supplemental Table 2. Primer sequences used in this study
